# Toll-Like Receptor 2 Mediates *In Vivo* Pro- and Anti-inflammatory Effects of *Mycobacterium Tuberculosis* and Modulates Autoimmune Encephalomyelitis

**DOI:** 10.3389/fimmu.2016.00191

**Published:** 2016-05-24

**Authors:** Alessia Piermattei, Giuseppe Migliara, Gabriele Di Sante, Maria Foti, Soren Bohos Hayrabedyan, Angela Papagna, Maria Concetta Geloso, Maddalena Corbi, Mariagrazia Valentini, Alessandro Sgambato, Giovanni Delogu, Gabriela Constantin, Francesco Ria

**Affiliations:** ^1^Laboratory of Immunology, Institute of General Pathology, Università Cattolica del Sacro Cuore, Rome, Italy; ^2^Department of Public Health and Infectious Diseases, University “La Sapienza”, Rome, Italy; ^3^Institute of Rheumatology, Università Cattolica del Sacro Cuore, Rome, Italy; ^4^Molecular Medicine and Immunology Laboratory, Genopolis Consortium, University of “Milano Bicocca”, Milan, Italy; ^5^Institute of Biology and Immunology of Reproduction, Bulgarian Academy of Sciences, Sofia, Bulgaria; ^6^Institute of Anatomy and Cell Biology, Università Cattolica del Sacro Cuore, Rome, Italy; ^7^Institute of General Pathology, Università Cattolica del Sacro Cuore, Rome, Italy; ^8^Institute of Microbiology, Università Cattolica del Sacro Cuore, Rome, Italy; ^9^Section of General Pathology, Department of Medicine, University of Verona, Verona, Italy

**Keywords:** TLR2, EAE/MS, host–pathogen interactions, inflammation, Foxp3

## Abstract

Mycobacteria display pro- and anti-inflammatory effects in human and experimental pathology. We show here that both effects are mediated by Toll-like receptor 2 (Tlr2), by exploiting a previously characterized Tlr2 variant (Met82Ile). Tlr2 82ile promoted self-specific proinflammatory polarization as well as expansion of ag-specific FoxP3^+^ Tregs, while Tlr2 82met impairs the expansion of Tregs and reduces the production of IFN-γ and IL-17 proinflammatory cytokines. Preferential dimerization with Tlr1 or Tlr6 could not explain these differences. *In silico*, we showed that Tlr2 variant Met82Ile modified the binding pocket for peptidoglycans and participated directly to a putative binding pocket for sugars and cadherins. The distinct pro- and anti-inflammatory actions impacted severity, extent of remission, and distribution of the lesions within the central nervous system of experimental autoimmune encephalomyelitis. Thus, Tlr2 has a janus function *in vivo* as mediator of the role of bacterial products in balancing pro- and anti-inflammatory immune responses.

## Introduction

The administration of adjuvants containing *Mycobacterium tuberculosis* (Mtb)-derived products that interact with pathogen-recognition receptors (PRRs) at the moment of antigen challenge is essential for the development of optimal allo- (1) and self-reactive immune responses (2–4). Yet, the administration of mycobacterium-derived products can promote or delay immunopathology, depending on disease type, timing of administration, and possibly genetic factors. Likewise, environment-derived agents contribute to the determination of autoimmune diseases, such as multiple sclerosis (MS), influencing the variability of clinical course and severity (5–9). In particular, the role of bacterial infections in modulating MS course is well established (10, 11), and it has been demonstrated that BCG administration (in human) or complete Freund’s adjuvant (CFA) (in mouse) is beneficial for the treatment of MS, delays the development of type I diabetes, and interferes with the development of allergy (12–18). Thus, products derived from mycobacteria have a janus role, being able to promote or downmodulate proinflammatory immune responses.

Pathogen-recognition receptors expressed by the distinct subsets of DC play a major role in determining the outcome of immune responses to pathogens, by regulating the secretion of directive cytokines (19). Among PRRs, Tlr2 is the main innate receptor recognizing products from Mtb, and it has the widest repertoire of ligands, co-acting with a variety of other molecules. Its main binding pocket for lipopeptides (and the selective synthetic ligands Pam2CSK4 and Pam3CSK4) lies between residue 286 and residue 366. No evidences have been reported about (a) different binding site(s) for those large, repetitive, and hydrophilic molecules that are also able to stimulate it.

Tlr2 regulates the secretion of directive cytokines, expression of costimulatory molecules and cell mobility. Tlr2 is expressed in many immune cell types, including antigen-activated T cells (20). Several authors have reported that Tlr2 contributes to the polarization toward proinflammatory phenotypes of T cells and controls Treg development (21–25) and function *in vitro*, increasing or decreasing it depending on the ligands used (26–28). However, it is not clear whether the opposite immunomodulatory effects of mycobacteria products can be mediated by Tlr2 also *in vivo*.

Tlr2 KO mice abrogate at the same time all signals coming from any Tlr2 dimer; since dimers of Tlr2 with distinct co-receptors may play different or even opposite roles in the development of the immune response when complex adjuvant, such as Mtb, is used, KO mice possibly do not represent a suitable model to assess the role of Tlr2 *in vivo*. Accordingly, it is not surprising that Tlr2 KO mice have provided conflicting evidences about its role *in vivo*, in EAE. In addition, genetic evidences show that also Tlr4 and Tlr9 are involved in the protection from Mtb, and Tlr4 is the second receptor for Mtb-derived motives (29). Thus, it is also possible that pro- and anti-inflammatory effects of Mtb may be mediated by distinct Tlrs.

We have previously shown that a single non-synonymous nucleotide polymorphism (SNP) exists between Tlr2 of SJL and B6 strains (generating a polymorphism Ile82Met, respectively). This polymorphism affects the sensitivity to the amount of Mtb in the adjuvant, leading to differences in the mobilization of activated T cells from the lymph nodes (LNs). Tlr2^82met^ displays a dominant ability over Tlr2^82ile^ to promote early exit of antigen-specific T cells in the presence of limiting amounts of Mtb (30). While Tlr2 expressed on the APCs mediates most effects of the engagement of Tlr2 on the immune response, it is the engagement of Tlr2 expressed by the activated T cells that determines the early/late mobilization from LNs (30).

Our model based on a Tlr2 polymorphism allows to examine the role of Tlr2 in F1 (SJL × B6^wt^) mice having Tlr2 of both SJL (Tlr2^82ile^) and B6 (Tlr2^82met^), with Tlr2^82met^ dominant, as well as F1 [SJL × B6^129TLR2tm 1 kir/3^(B6^Tlr2−^)] mice displaying only Tlr2^82ile^ of SJL origin (30). We have previously shown that the two F1 mice express similar Tlr2 levels on immune cells, despite having a different gene dosage. This model is less intrusive than a KO model and allows defining as Tlr2-dependent those functions that are differentially expressed *in vivo* in the above described F1 mice, leaving Tlr2 intact.

Notably, both parental strains SJL and B6 develop EAE, but with distinct clinical courses (31, 32), suggesting that Tlr2 polymorphism may impact the disease development in these mice (33–36). Thus, this model is also a good candidate to define if and how Tlr2 represents a path for infectious agents to modulate pro- and anti-inflammatory autoimmunity.

We hereinafter report that Tlr2 haplotype regulates the secretion of type 1/17 cytokines and Treg polarization. We found significantly higher levels of basal and antigen-driven production of proinflammatory cytokines IFN-γ and IL-17 in F1 (SJL × B6^Tlr2−^) mice. Also, the level of mRNA specific for FoxP3 and the number of CD4^+^CD25^+^FoxP3^+^ cells was increased by antigen stimulation in F1 (SJL × B6^Tlr2−^) mice, but not in F1 (SJL × B6^wt^) mice. Both pro- and anti-inflammatory effects of the polymorphism appeared due to the modification of the secretion of polarizing cytokines produced by the APCs. When we studied *in silico*, the effect of the polymorphism at position 82 on Tlr2, we observed that this residue is directly involved in a secondary pocket binding several sugars and cadherins, and influences the primary binding pocket of Tlr2. Furthermore, we found significant differences in the course and pathology of EAE in F1 (SJL × B6^wt^) vs. F1 (SJL × B6^Tlr2−^) mice.

Thus, through the mediation of both pro- and anti-inflammatory effects of Mtb, Tlr2 is the molecular link between infectious history and autoimmune disease course, and a potential point of therapeutic intervention.

## Materials and Methods

### Ethics Statement

This study was carried out in accordance with the recommendations of Ethic Committee for Animal Experimental of Università Cattolica del Sacro Cuore. All the experiments were performed in accordance with the experimental protocol sf 46373 (II25) approved by the Ethic Committee for Animal Experimental of Università Cattolica del Sacro Cuore.

### Mice and Peptides

The 8- to 10-week-old SJL, C57Bl/6 (B6^wt^) mice and B6 129^TLR2tm 1 kir/3^(B6^Tlr2−^) mice were purchased from Charles River (Calco, Italy) and kept in a conventional facility at the Catholic University of the Sacred Heart of Rome.

SJL and B6 mice were crossed to obtain F1 (SJL × B6^wt^) mice that have Tlr2 of both SJL and B6, whereas SJL and B6^Tlr2−^ were crossed in order to obtain F1 (SJL × B6^Tlr2−^) mice that have only the copy of Tlr2 of SJL origin.

Tlr2 heterozygous F1 (SJL × B6^Tlr2−^) mice with one copy of Tlr2 of SJL origin, and Tlr2 homozygous F1 (SJL × B6^*ts*^) mice, with two copies of Tlr2 of SJL origin, were obtained by crossing heterozygous (B6^−/*ts*^) mice with SJL mice, as previously described (21). Briefly, B6^−/*ts*^ mice were obtained back-crossing F1 (SJL × B6^Tlr2−^) mice to B6^Tlr2−^ mice for nine further generations.

Peptide 139–151 (HSLGKWLGHPDKF) (p139) of the proteolipid protein was purchased from PRIMM (Milan, Italy) and was >95% pure, as determined by HPLC and mass spectroscopy.

The TLR2 agonists Pam2CSK4 (tlrl-pm2 s-1) and Pam3CSK4 (tlrl-pms) were used at different concentrations, following the recommendations of the supplier (Invivogen, San Diego, CA, USA): for cell stimulation at 100 ng ml^−1^ and for immunization at 10 μg/mouse combined with incomplete Freund’s adjuvants (IFAs) as described below.

### Immunization

Mice were immunized subcutaneously (s.c.) with 50 μg/mouse of p139 in PBS emulsified 1:1 with enriched CFA IFA containing 4 mg/ml killed and heat-dried *M. tuberculosis* H37RA (Sigma-Aldrich, St. Louis, MO, USA) in a final volume of 100 μl/mouse. Draining LNs were collected from mice 4 or 10 days after immunization. For cytokines and nuclear factors assessment, 5 × 10^5^ lymph node cells (LNC) per well were cultured in presence or absence of p139 10 μg/ml for 3 h in RPMI-1640 medium (Sigma-Aldrich, St. Louis, MO, USA) supplemented with 2 mM l-glutamine, 50 μM 2-ME, 50 μg/ml gentamicin (Sigma-Aldrich, St. Louis, MO, USA), and 0.2% mouse serum. LNC were resuspended in RLT buffer for RNA extraction. For flow cytometry and for cytokines production assessment, LNC were cultured for 18 h at the same conditions described above.

### Microarray Analysis

#### Total RNA Extraction

Total RNA was extracted from six samples [three F1 (SJL × B6^wt^) and three F1 (SJL × B6^Tlr2−^) mice] in total using TRIZOL^®^ reagent (Life Technologies Inc., Carlsbad, CA, USA) and then purified with RNeasy Micro columns (Qiagen, Venlo, Netherlands), as described by the manufacturers. The quality of all samples was strictly controlled to verify the RNA integrity before use in microarray experiments. RNA quantity and purity were evaluated spectrophotometrically by Nanodrop 1000 (Thermo Scientific, Waltham, MA, USA) and then by Quant-iT™ Ribogreen^®^ RNA Assay kit (Invitrogen, Waltham, MA, USA), while the quality was assessed by the Agilent 2100 bioanalyzer RNA Pico 6000 kit (Agilent Technologies, Inc., Santa Clara, CA, USA). Only samples with good RNA yield and no RNA degradation (28S:18S >1.7 and RNA integrity number >6) were retained for further experiments.

#### Gene Expression Microarray

One hundred nanograms of each total RNA was processed using the Ambion^®^ WT Expression kit (Life Technologies, Waltham, MA, USA) with T7-Oligo(dT) Primers, adding in the mix 2 μl of poly-A RNA controls (*lys*, *phe*, *thr*, and *dap*). These controls were amplified and labeled together with the total RNA sample. The 10 μg of cRNA were input into the second cycle cDNA (ss-cDNA) reaction, and then, 5.5 μg of ss-cDNA were input for fragmentation, using the GeneChip^®^ WT Terminal Labeling kit (Affymetrix, Santa Clara, CA, USA). Each cDNA fragment was end labeled with biotin using terminal deoxynucleotidyl transferase (37) before being hybridized to the arrays and, finally, was added to a hybridization cocktail (50-pM control oligonucleotide B2; 15, 5, 25, and 100 pM, respectively, of *BioB*, *BioC*, *BioD*, and *cre* hybridization controls; 1× hybridization mix; 10% DMSO; nuclease-free water): this mixture was heated to 99°C for 5 min and to 45°C for another 5 min. Then, it was injected into Affymetrix Mouse Exon 1.0 ST microarray chips (Affymetrix, Santa Clara, CA, USA), and the hybridization was performed under rotation at 45°C ± 16 h. The GeneChip^®^ Mouse Exon 1.0 ST Array is a single array with over 4.5 million unique 25-mer oligonucleotide features constituting approximately 1.2 million probe sets. For the gene-level analysis, the chip contains about 23339 probe sets.

After hybridization, the arrays were washed and stained with R-phycoerythrin streptavidin in the GeneChip^®^ Fluidics Station 450 and then were scanned with the GeneScanner 3000 System. Affymetrix GeneChip^®^ Command Console (AGCC) and Expression Console software were used for washing, scanning, and basic analysis of the arrays that produced .CEL intensity files. Following scanning, array images were assessed by eye to confirm scanner alignment and the absence of significant bubbles or scratches. BioB spike controls were found to be present on 100%, with BioC, BioD, and CreX also present in increasing intensity. Moreover, the hybridization intensities of poly-A controls helped to monitor the labeling process, independently from the quality of the starting RNA samples.

#### Bioinformatics Analysis

The following analysis steps were performed using Partek Genomic Suite 6.6 (Partek Inc., St. Louis, MO, USA) and Transcriptome Analysis Console (TAC) 3.0 (Affymetrix) software. Three quality checks were performed to verify the quality of sample preparation and hybridization.

The pos_vs_neg_AUC value: this is the area under the curve (AUC) for a receiver operating characteristic (ROC) plot comparing the signal values for positive controls with those for negative controls. The ROC curve is generated by evaluating how well the probe set signals separate the positive controls from the negative ones with the assumption that the negative ones are a measure of false positives and the positive controls are a measure of true positives. An AUC of 1 reflects perfect separation, whereas an AUC value of 0.5 would reflect no separation. Values between 0.80 and 0.90 are typical.

The principal component analysis (PCA) for all samples was used to verify the distributions of the replicates: this multivariate technique allows reduction of the high dimensionality of genes space. In this way, samples and genes can be represented in multiple bidimensional spaces, and plotted to visually assess similarities and differences between samples and determine whether samples can be grouped. Outlier samples should be identified and eliminated. This is particularly true in analysis of exon array data since low sample quality is likely to be highly influential in generating noise, therefore leading to high false positive rates (38). Hierarchical clustering based on complete linkage method and Pearson correlation as similarity measure was applied to evaluate the effect of the different sources of variability (sample and host specific responses). The resulting dendogram can be interpreted similarly to a phylogenetic tree.

**Figure 1:**
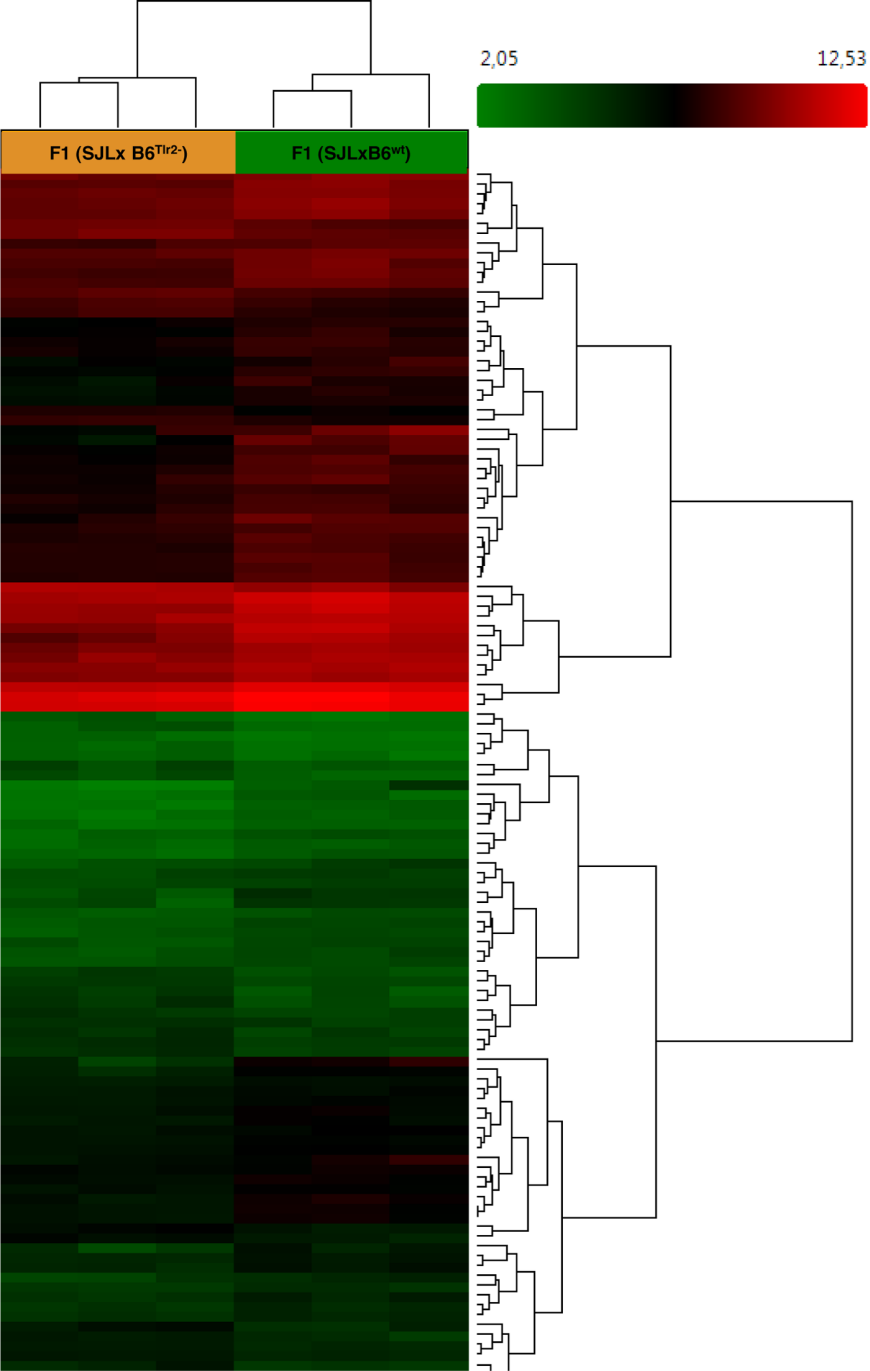
**Microarray analysis indicates that Tlr2 genotype modulates immune-related pathways**. Three F1 (SJL × B6^wt^) and three F1 (SJL × B6^Tlr2−^) were immunized s.c. with CFA as described. The mice were sacrificed at day 4 after immunization. Heat map showing different expression patterns of the most significant regulated genes, selected with a fold change <1.5 and >1.5 and a *p*-value of 0.05 in the F1 (SJL × B6^Tlr2−^) and F1 (SJL × B6^wt^) samples. The heat map indicates upregulation (red), downregulation (green), and mean gene expression (black). The columns represent individual samples, while the rows represent the genes. A total of 23339 genes in gene-level analysis were tested.

After these quality checks, based on PCA shown in Figure 1, six samples (three samples for condition) were used for the final analysis. Affymetrix Expression Console was used to process the original .CEL files and generate .chp files using the RMA-sketch workflow after signal summarization (median polish) (39) and data normalization (sketch-quantile method).

A gene-level analysis was performed by TAC software: a total of 23339 genes were tested to compare their expression between 3 F1 (SJL × B6^Tlr2−^) samples and 3 F1 (SJL × B6^wt^) samples. The analysis of variance (ANOVA) was used for the detection of differentially expressed genes (DEGs). Only the probe sets whose fold changes were higher than or equal to 1.5 and *p* value of 0.05 were selected as modulated.

#### Cytokine Production

Medium from cultures were collected and stained with MACSPlex Cytokine 10 Kit (Miltenyi Biotec, Bergisch Gladbach, Germany), following the manufacturer’s protocol. The samples were read using the Navios flow cytometer (Beckman Coulter, Pasadena, CA, USA) and analyzed with Kaluza Analysis 1.3 software (Beckman Coulter, Pasadena, CA, USA). Samples were performed as duplicate.

#### Cytokine and Transcription Factors mRNA Measurement by Quantitative Real-Time PCR

Measurement of cytokines mRNA was performed on LN cells and infiltrating cells from the CNS. Total mRNA was isolated by RNeasy Mini Kit and cDNA was synthesized using an oligo-dT primer (dT15) according to the manufacturer’s instructions (Qiagen, Valencia, CA, USA). The optical density of extracted mRNA was determined at 260 nm using a NanoDrop 1000 (Thermo Scientific, Waltham, MA, USA).

Quantitative RT-PCRs were performed with iQ™SYBR^®^ Green Supermix and the iCycler instrument (Bio-Rad, Hercules, CA, USA). Relative expression levels of cytokine and transcription factors mRNAs were normalized by 18S as a housekeeping gene and calculated by the 2^−ΔΔCt^ method (40).

Primers sequences (Invitrogen™ Life Technologies, Paisley, UK) are listed in Table S2 in Supplementary Material in the 5′–3′ orientation. Once the reaction conditions were settled, the samples were loaded as triple replicates.

#### Determination of CD4^+^CD25^+^FoxP3^+^ Cell

Lymph node cells from F1 (SJL × B6^wt^) and F1 (SJL × B6^Tlr2−^) were cultured as described above.

CD4^+^CD62L^high^ and CD4^+^CD62L^low^ T cells were enriched from the spleens of naive SJL using CD4^+^CD62L^+^ T Cell Isolation Kit II (Miltenyi Biotec, Bergisch Gladbach, Germany), according to the manufacturer’s instructions. Due to low yield, CD4^+^CD62L^high^ cells obtained from different mice of the same groups were pooled together. Cells were cultured for 6 h in medium (as described above) in presence or absence of 100 ng/ml of Pam2CSK4 or Pam3CSK4 (Invivogen^®^, San Diego, CA, USA), with or without Anti-biotin MACSBead Particles conjugated with αCD3ϵ + αCD28 (T cell Activation/Expansion kit MACS^®^ Miltenyi Biotec, Bergisch Gladbach, Germany), following the manufacturer’s protocol. The same conditions were repeated in presence or absence of anti-galectine 9 (ab127982, dilution 1:1000, rabbit polyclonal, Abcam, Cambridge, UK) at 0.1 μg/ml.

After harvesting, the cells were stained using the Treg Detection Kit (Miltenyi Biotec, Bergisch Gladbach, Germany). The cells were tested using the Navios flow cytometer (Beckman Coulter, Pasadena, CA, USA) and analyzed with Kaluza Analysis 1.3 software (Beckman Coulter, Pasadena, CA, USA).

### Bioinformatics Structural Analysis

#### Ligand-Binding Analysis

Functional annotation of TLR2 was initially done using ligand-binding site prediction based on COFACTOR algorithm (41).^1^ The 3D structural model template-based ligand–protein docking uses consensus-based algorithm COACH for binding site prediction and the semi-manually curated database BioLiP for biologically relevant ligand–protein interactions, containing more than 204.223 entries, each having annotations on ligand-binding residues, ligand-binding affinity, catalytic sites, Enzyme Commission numbers, Gene Ontology (GO) terms, and cross-links to other databases.

#### Molecular Dynamics of Tlr2

A crystallography model of Tlr2 complexed with Tlr6 and having a ligand Pam2CSK4 (PDB 3A79) was used for Tlr2^82ile^ as it was expressed to have the sequence found in the SJL strain. The I-Tasser server (42) for template-based modeling was used as best in class for homology combined with *ab initio* modeling to produce relevant initial structure for Tlr2 of B6 (where Met was substituted in place of Ile). Model preprocessing for missing H-bond addition and steric classes was done using the built-in algorithms of PDB2PQR and MDWeb. The ligand and other HETAMs were removed during this process. A complete setup for AMBER Parm99SB* forcefield (structure minimization, solvation with TIP3P water molecules, and 50-mM ions and equilibration using NPT ensemble with periodic boundary conditions) was made using MDWeb server (43).^2^ The equilibrated structural models were also used for an exploration of individual anisotropic normal mode localized perturbations, based on Protein Energy Landscape Exploration (PELE normal mode analysis) algorithm.^3^ The latter combines a Monte Carlo stochastic approach with protein structure prediction (44).

#### Secondary Structure Analysis and Trajectory Analysis

The obtained results by PELE normal mode analysis trajectory were analyzed using Timeline plugin of VMD^4^ for following the temporally changing per-residue attributes of a molecular structure such as RMSd and residue displacement (45). The H-bonds were calculated per-frame using Chimera script in MD trajectory analysis module (46).

#### Ligand-Binding Interface Volume Analysis

Pam2CSK4 docked to Tlr2^82met^ or Tlr2^82ile^ was subject to ligand-docked volume analysis using Chimera per-frame script as well as using MOLE2 algorithm for ligand-binding cavity detection (47). The obtained data were plotted in Excel.

#### EAE Induction and Clinical Evaluation

The 8- to 10-week-old female mice were immunized s.c. with 75 μg/mouse of p139 in PBS emulsified 1:1 in enriched CFA (100 μl/mouse). The 300 ng of Pertussis toxin (List Biological Laboratories, Inc., Campbell, CA, USA) were injected intraperitoneally on the day of immunization and 3 days later. Clinical signs of EAE were evaluated daily and in a blinded fashion according to the following scale: 0, no clinical score; 1, loss of tail tone; 2, weak hind leg paresis; 3, posterior leg paresis; 4, complete paraplegia; and 5, death (48, 49). Intermediate values were used for incomplete symptoms.

Mice at disease peak were perfused in PBS and sacrificed under deep anesthesia (ketamine 75 mg/kg and medetomidine 1 mg/kg i.p.). CNS-infiltrating cells were isolated by Percoll gradient and were resuspended in RLT buffer for RNA extraction.

#### CNS Histology

Mice, under deep anesthesia (ketamine 75 mg/kg and medetomidine 1 mg/kg i.p.), were sacrificed and perfused through the aorta with 50-ml saline solution, followed by 50 ml of 0.01M, pH 7.4 PBS, and 4% paraformaldehyde (VWR International, Radnor, PA, USA). Brains and spinal cords were removed and immersed in the same fixative for 24 h. The brains were cut into different blocks at three different levels along the rostro-caudal axis: frontal lobes, hippocampus/thalamus, and cerebellum/brain stem, respectively, taken from the following bregma coordinates: from +2.22 mm to +0.38 mm, from −1.22 mm to −2.80 mm, and from −2.92 mm to −6.64 mm according to the atlas of Paxinos and Franklin (50) and then embedded in paraffin. Serial 10-μm coronal sections from the brain and spinal cord were cut on a microtome and then processed for histological analysis (H&E) to assess for inflammatory lesions or immunocytochemistry to reveal inflammatory cells (CD3-expressing lymphocytes and activated microglia), as previously described (4). Briefly, for CD3 labeling, sections were incubated overnight at +4°C with the primary Ab (rat monoclonal anti-CD3; BD Biosciences, Franklin Lakes, NJ, USA) and then for 1 h with a biotin-conjugated secondary Antibody (Vector Laboratories, Burlingame, CA, USA). Microglia cells were identified with the specific histochemical marker Ricinus Communis Agglutinin I (biotinylated Ricinus Communis Agglutinin 120; Vector Laboratories). Immunoreactive cells were visualized by the avidin–biotin immunoperoxide method with diaminobenzidine as chromogen (Vectastain Elite ABC Kit; Vector Laboratories, Burlingame, CA, USA).

Histological scores of infiltrates were assessed using a semiquantitative method, essentially as described also by other groups (51). Briefly, regularly spaced (every 100 μm) brain and spinal cord sections were analyzed with a brightfield microscope equipped with an AxioCam MRc and AxioVision software (Axiophot, Zeiss, Milan, Italy), at a magnification of 20×. Sections were photographed and blindly evaluated according to the following criteria: 0 = no inflammation; 1 = cellular infiltrates only around blood vessels and meninges; 2 = moderate cellular infiltrates in parenchyma, less than 50% of the white matter (WM); and 3 = severe infiltrates in parenchyma, deep and/or more than 50% of the WM.

#### Statistics

Statistics were performed with two-tailed Mann–Whitney test, one-way ANOVA with Bonferroni’s multiple comparison test, two-tailed Wilcoxon matched-pairs signed rank test, or χ^2^ test. *p* < 0.05 was considered significant (48, 49). Data were analyzed with GraphPad Prism 5.03 software (GraphPad Software, Inc., La Jolla, CA, USA).

## Results

### Microarray Analysis of LN Cells Shows That Tlr2 Polymorphism Modulates Immune-Related Pathways, Despite Overall Remarkably Similar Responses

We have previously demonstrated a role for Tlr2 in T cell mobilization in the presence of limiting amounts of Mtb in the adjuvant. To further understand the impact of Tlr2 polymorphism on autoimmunity and inflammation, we compared global gene usage of enriched LN-derived T cells from F1 (SJL × B6^Tlr2−^) mice displaying only Tlr2^82ile^ of SJL origin, and F1 (SJL × B6^wt^) having Tlr2 of both SJL (Tlr2^82ile^) and B6 (Tlr2^82met^) strains, with Tlr2^82met^ dominant. We performed the comparison 4 days after challenging mice with CFA containing 50 μg/mouse of Mtb s.c., a time point at which we had shown that all primed antigen-specific T cells were still in the draining LN.

A total of 23339 genes were tested at core level to compare their expression between the two groups of F1 (SJL × B6^Tlr2−^) and F1 (SJL × B6^wt^) mice. As expected, due to high genetic homology between the F1 mice, 35 genes were found to be differentially expressed with absolute fold change >2 and ANOVA *p*-value <0.05 [one-way between-subject ANOVA (unpaired) method] and 125 genes with absolute fold change >1.5 and *p*-value <0.05 (52). The heat map for the 125 genes is displayed in Figure 1.

To reveal global functional differences, the GO hierarchy analysis was then carried out on the DEGs. The genes were categorized according to the WikiPathways in TAC software. This software allowed selecting the principal pathways showing a modulation dependent on the Tlr2 haplotype (Table 1).

**Table 1:** **Principal pathways modulated in F1 (SJL × B6^Tlr2−^) compared to F1 (SJL × B6^wt^)**.

Pathway	#Total	#Up	Up list	#Down	Down list
B cell receptor signaling pathway	11	0		11	Lyn, Syk, Blnk, Cr2, Cd19, Pik3ap1, Cd22, Fcgr2b, Cd72, Cd79a, Rasgrp3
XPodNet – protein–protein interactions in the podocyte expanded by STRING	5	0	Sh3bp1, Tspan7, Adam12	5	Cd19, Cd22, Blnk, Lyn, Slc4a1
IL-2 signaling pathway	4	0		4	Il2, Syk, Socs3, Lyn
Adipogenesis genes	4	1	Scd1	3	Socs3, Ebf1, Mef2c
IL-3 signaling pathway	4	0		4	Lyn, Syk, Socs3, Fcer2a
Cytokines and inflammatory response (BioCarta)	4	0		5	Il10, Il13, Il2, H2-Eb1
Odorant GPCRs	4	2	Gpr173, Tas2r138	2	Taar7d, Vmn2r1
Metapathway biotransformation	4	1	Cyp1a1	3	Cyp4f18, Chst3, Hs3st1
Spinal cord injury	3			3	Fcgr2b, Il2, Klk8
Chemokine signaling pathway	3	0		3	Ccl3, Lyn, Gm11787
T cell receptor signaling pathway	2	0		2	Syk, Lyn
Inflammatory response pathway	2	0		2	Il2, Cd86
IL-6 signaling pathway	2	0		2	Socs3, Lyn

The most relevant pathways are lymphocytes signaling, cytokines, and inflammatory response-related pathways. It is of interest that also the pathway linked to spinal cord injury appears modulated by the polymorphism at position 82 of Tlr2.

Using this analysis, it was immediately noted that Tlr2^82ile^ [i.e., the response observed in F1 (SJL × B6^Tlr2−^) mice] is associated with a downmodulation of the pathways linked to type 2 cytokines. The cytokines IL-10, IL-13, IL-5, and IL-2 were all significantly down regulated in F1 (SJL × B6^Tlr2−^) mice compared to F1 (SJL × B6^wt^) mice (Table S1 in Supplementary Material). Therefore, the transcriptomics data strongly suggested that the F1 (SJL × B6^Tlr2−^) mice display an aberrant deregulation of the T cells skewing pathways (Figure S1A in Supplementary Material). Additional analysis of the transcriptomics data for T cells skewing pathways genes for the Th17 lineage induction was than performed. The genes for IL-17f, IL-22, and Rorc were upregulated in F1 (SJL × B6^Tlr2−^) mice, although with a *p*-value higher than 0.05 (Figure S1B in Supplementary Material). The T cells transcriptomics data from the F1 (SJL × B6^Tlr2−^) mice suggested that these mice may be prone to develop a proinflammatory phenotype already at day 4 after CFA administration. Therefore, based on these findings, we decided to study the Th cell differentiation program in our mouse model to understand the impact of the Tlr2 polymorphism on T cells polarization during CFA immunization and EAE induction.

### Tlr2^82ile^ Promotes the Bias toward Type 1/17 of p139-Specific Response in Lymph Node Cells

**Figure 2:**
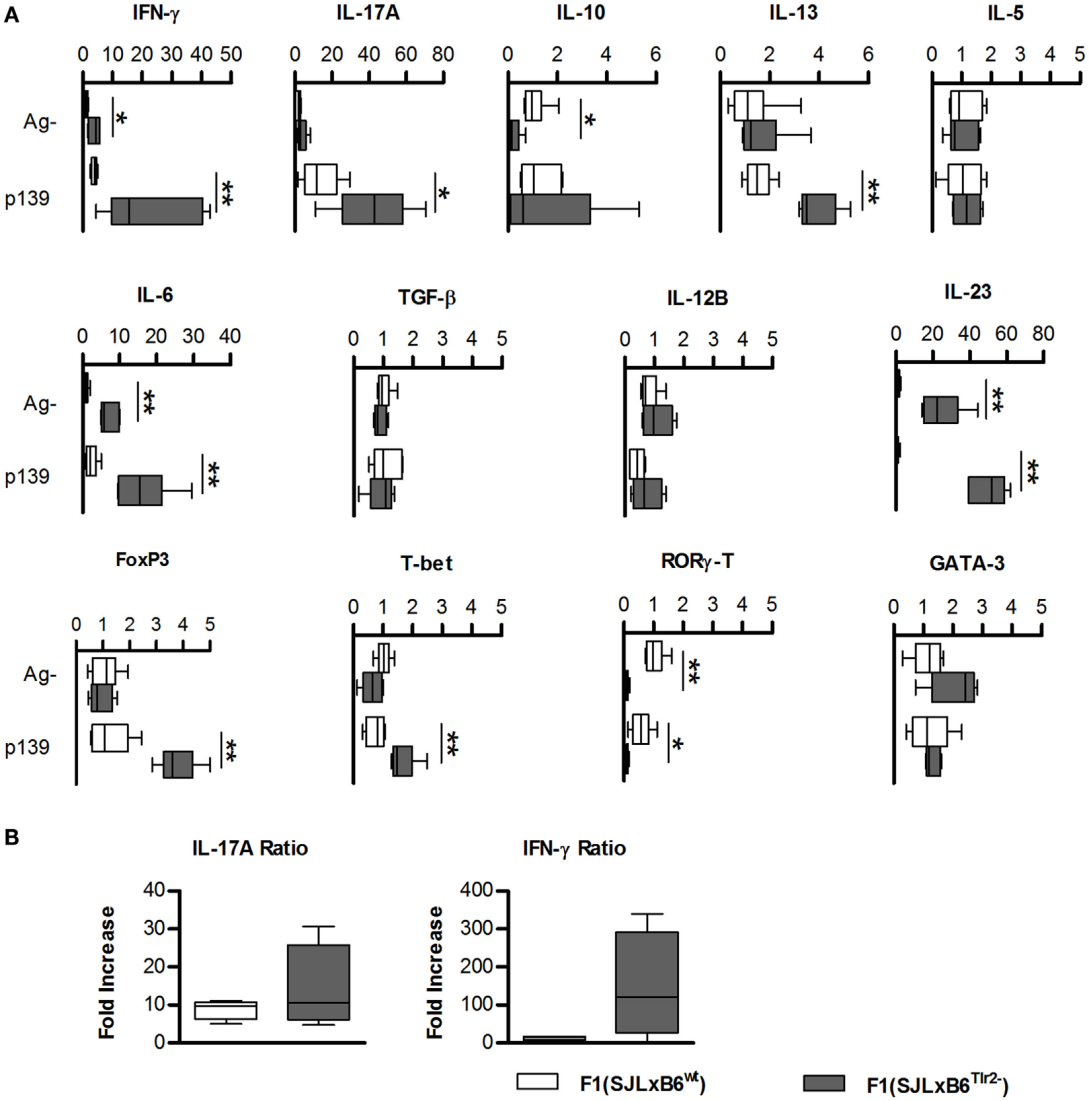
**Tlr2 genotype regulates cytokines and T cell nuclear factors**. **(A)** Groups of six F1 (SJL × B6^wt^) (open bars) and six F1 (SJL × B6^Tlr2−^) (closed bars) mice were immunized s.c. with p139 in enriched CFA. Each group of F1 mice was composed of three mice of two distinct litters. Lymph node cells were cultured for 3 h with or without p139 as described in Section “Materials and Methods.” Effect of antigen stimulation on the levels of the mRNAs from cells obtained from LN draining the site of immunization in F1 (SJL × B6^wt^) (open symbols) and F1 (SJL × B6^Tlr2−^) (closed symbols) specific for the cytokines: IFN-γ, IL-17A, IL-10, IL-13, IL-5, IL-6, TGF-β, IL-12b, and IL-23, and for nuclear transcription factors: FoxP3, T-bet, RORγ-T, and GATA-3. Levels of mRNA specific for the cytokines and for the transcription factors mentioned above were analyzed by qRT-PCR as described in Section “Materials and Methods” and reported as fold increase (boxplot) after normalization of the amount of each specific mRNA vs. the average value obtained from the unstimulated LNC of F1 (SJL × B6^wt^) mice (two-tailed Mann–Whitney test, *p < 0.05, **p < 0.01). qRT-PCR reactions were performed in triplicate for each sample from mice of both groups. **(B)** Groups of six F1 (SJL × B6^wt^) (open bars) and six F1 (SJL × B6^Tlr2−^) (closed bars) mice from two distinct experiments were immunized s.c. with p139 in enriched CFA. Lymph node cells were cultured for 18 h with or without p139 as described at Section “Materials and Methods.” The graphics reports the effect of antigen stimulation on the production of IL-17A and IFN-γ as ratio between p139 and Ag- (two-tailed Mann–Whitney test, *p < 0.05). The cytokines quantification was performed by Macsplex^®^ as described in Section “Materials and Methods.”

To confirm the observations obtained from microarray analysis suggesting that the Tlr2 polymorphism is able to modulate the balance of Th polarization and to examine its influence in an antigen-specific response, we next quantified the amount of mRNAs specific for cytokines produced by T cells and by antigen-presenting cells (APCs), and for lineage-specific nuclear transcription factors in unstimulated or p139-stimulated LNC (Figure 2A). We observed that the presence of Tlr2^82ile^ alone in F1 (SJL × B6^Tlr2−^) mice (full bars) resulted in a significantly higher basal level (*p* < 0.05) of the mRNAs specific for IL-6, IL-23, and IFN-γ, whereas it decreased significantly the basal levels of mRNAs for IL-10 and RORγ-T compared to the levels observed in LNC from F1 (SJL × B6^wt^) mice (open bars). Although the level of mRNA specific for IL-17A also appeared increased in mice having only Tlr2^82ile^, in this latter case, the difference fell short of statistical significance (*p* = 0.17).

Following antigen stimulation, LNC from F1 (SJL × B6^Tlr2−^) mice showed a further increase of the levels of mRNAs specific for IL-6, IL-23, and IFN-γ. In addition, also the level of mRNA specific for IL-17A was significantly higher in the presence of Tlr2^82ile^ alone after antigen challenge.

The presence of Tlr2^82ile^ alone increased the expression of T-bet after p139 stimulation, which was consistent with the higher levels of IFN-γ specific mRNA, despite the fact that we observed only mild differences in levels of mRNA specific for IL-12b in either experimental condition. There were no significant differences in the levels of mRNA specific for RORγ-T (despite the presence of higher values of mRNAs for IL-6, IL-23, and IL-17A) and GATA-3 under basal conditions or following stimulation with p139.

In apparent contrast with the observation of the proinflammatory attitude of immune LNC in mice carrying only Tlr2^82ile^, we found that LNC from F1 (SJL × B6^Tlr2−^) mice also upregulated FoxP3 to a significant higher level than LNC from F1 (SJL × B6^wt^) mice, after antigen stimulation.

We next examined the levels of cytokines secreted by LNC. Cells obtained from F1 (SJL × B6^Tlr2−^) mice produced higher levels of IL-17A and antigen-driven secretion of IFN-γ (expressed as the ratio between values obtained after stimulation with the ag/values in unstimulated cells) was also higher in F1 (SJL × B6^Tlr2−^) mice (Figure 2B). In addition, the amount of type 2 cytokines was similar in both groups with no antigen-dependent secretion of IL-4, IL-5, and IL-10 (Figure S2 in Supplementary Material). Thus, Tlr2^82ile^ promoted the polarization of T cell-dependent response toward type 1/17, whereas this effect was dampened by the presence of the allele 82met. The levels of IL-12 and IL-23 were below the detection limit, and we could not determine if the difference in the secretion of cytokines by T cells was associated also with similar alterations of the secretion of directive cytokines by APCs, as suggested by Q-PCR data.

### Tlr2^82ile^ Promotes the Expansion of Antigen-Specific CD4^+^CD25^+^FoxP3^+^ Cells

The results described above suggest that Tlr2^82ile^ promotes the expression of the mRNA specific for FoxP3 upon antigen stimulation, despite inducing at the same time an increase of IFN-γ and IL-17A. Thus, our next aim was to establish if polymorphism at position 82 of Tlr2 has an effect on the number of antigen-specific CD4^+^CD25^+^FoxP3^+^ population.

**Figure 3:**
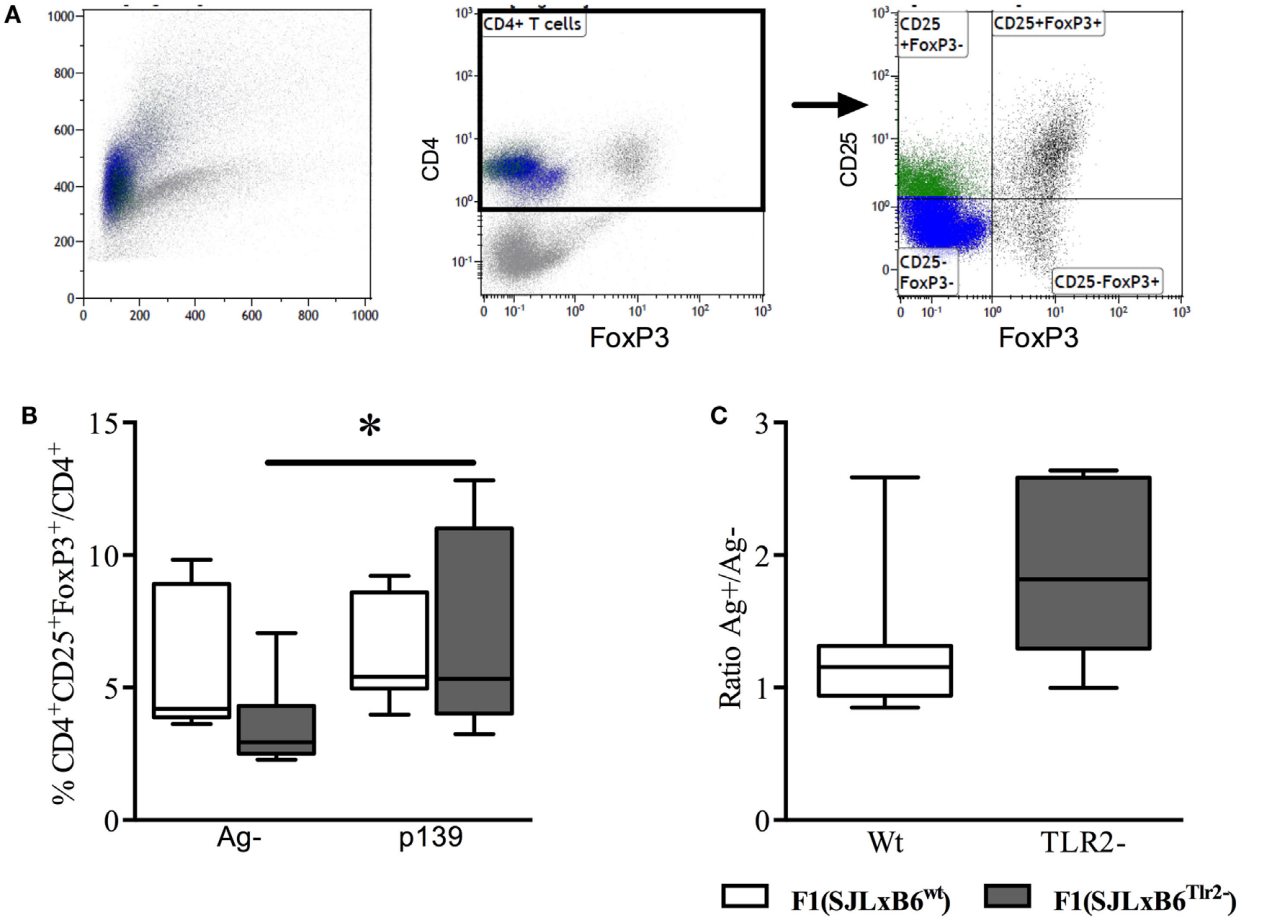
**Tlr2 genotype regulates Treg levels**. Ten F1 (SJL × B6^wt^) (open bars and symbols) and nine F1 (SJL × B6^Tlr2−^) (closed bars and symbols) mice from three distinct experiments were immunized s.c. with p139 in enriched CFA. The mice were sacrificed at day 12 after immunization. Lymph node cells were cultured for 18 h with or without p139 as described in Section “Materials and Methods.” Cells were stained for CD4, CD25, and FoxP3, and analyzed by flow cytometry. **(A)** Gating of CD4^+^CD25^+^FoxP3^+^ cells; **(B)** percentage (boxplot) of CD4^+^CD25^+^FoxP3^+^ cells upon total CD4^+^ cells and as **(C)** ratio (boxplot) between percentages of CD4^+^CD25^+^FoxP3^+^ cells upon total CD4^+^ in stimulated and unstimulated cells per group (two-tailed Wilcoxon matched-pairs signed rank test *p < 0.05).

To this end, we immunized F1 mice with p139 and established the frequency of CD4^+^CD25^+^FoxP3^+^ cells among total CD4^+^ LN-derived cells by flow cytometry. The results showed that the stimulation with p139 significantly increased the frequency of CD4^+^CD25^+^FoxP3^+^ cells in F1 (SJL × B6^Tlr2−^) mice, but not in F1 (SJL × B6^wt^) mice (Figure 3). No effect of the polymorphism could be observed when we examined the levels of CD4^+^CD25^−^FoxP3^+^ or CD4^+^CD25^+^FoxP3^−^ cells. Thus, the allele Tlr2^82ile^ promoted, whereas the allele Tlr2^82met^ dominantly dampened the expansion of FoxP3^+^CD4^+^ cells in response to antigen.

Together, these data suggest that allele Tlr2^82ile^ promotes pro- and anti-inflammatory immune responses, whereas Tlr2^82met^ dominantly downmodulates polarization to Type1/17 and Treg cells.

### Activation of Tlr2 by Pam2CSK4 or Pam3CSK4 on CD62^high^ or CD62^low^ T Cells Does Not Modify the Number of CD4^+^CD25^+^FoxP3^+^ Cells

**Figure 4:**
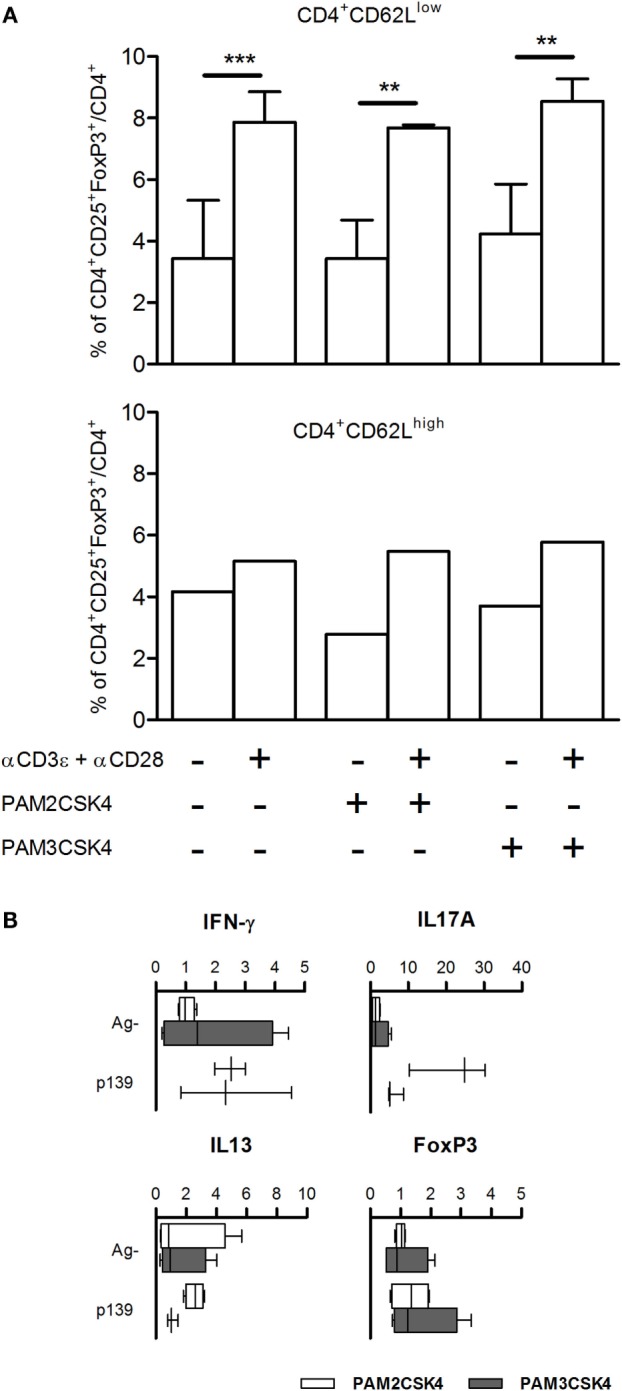
**(A)** FoxP3 levels are not regulated by Tlr2 expressed on T cells. CD4^+^CD62L^low^ and CD4^+^CD62L^high^ T cells were enriched from spleen cells of five naive SJL mice as described in Section “Materials and Methods.” CD4^+^CD62L^high^ cells were pooled together. The cells were cultured for 18 h with or without Pam2CSK4 or Pam3CSK4 and with or without αCD3ϵ + αCD28. Cells were stained for CD4, CD25, and FoxP3, and analyzed by flow cytometry. Results are showed as percentage + SD of CD4^+^CD25^+^FoxP3^+^ cells on total CD4^+^ cells (one-way ANOVA, Bonferroni’s multiple comparison test **p < 0.01; ***p < 0.001). **(B)** Pam2CSK4 or Pam3CSK4 as adjuvants do not modulate cytokines and T cell nuclear factor FoxP3. Eight female SJL mice were immunized s.c. with p139 in IFA mixed with Pam2CSK4 (four mice, open bars) or Pam3CSK4 (four mice, closed bars). Draining lymph node cells were cultured for 6 h with or without p139 as described in Section “Materials and Methods.” The graphics report the effect of antigen stimulation on the production of cytokines (boxplot). Cytokines quantification was performed by SybrGreen qPCR.

We have previously demonstrated that the effect of polymorphism Ile82Met on T cell exit from LNs is due to Tlr2 expressed on T cells. It had been previously reported that Tlr2 is expressed mainly on CD4^+^CD25^+^ T cells; its ligation by HSP60 on purified Tregs leads to an increase in the regulatory ability of Treg cells (26), while others showed conflicting evidences about effects of ligation of Tlr2 by Pam3CSK4 in purified Tregs (27, 28). To address the role of Tlr2 expressed on T cells in Treg polarization, we isolated CD62^high^ and CD62^low^ T cells from naive SJL mice, stimulated them with Pam2CSK4 or Pam3CSK4, in the absence or presence of αCD3ϵ + αCD28. The results (Figure 4A) showed that treatment with αCD3ϵ + αCD28 expanded the number of CD4^+^CD25^+^FoxP3^+^ cells, mainly in the CD62^low^ T cells, thus confirming in the parental strain SJL (Tlr2^82Ile^) the observations reported in Figures 2 and 3 for the ag-driven response in LNC of F1 mice (SJL × B6^Tlr2−^). Ligation of Tlr2 did not modify such an expansion. It was previously shown that CD44/alpha-galectin 9 is involved in the regulation of Treg polarization. However, we found no effect on Treg expansion of the presence of anti-galectin 9 antibodies (data not shown). Taken together, these data contrast with the hypothesis that the modulation of Th polarization by Tlr2 haplotype observed is due to the effect of ligation of Tlr2/Tlr1 or Tlr2/Tlr6 expressed by T cells, following or during T cell priming.

### Pam2CSK4 or Pam3CSK4 as Adjuvants Does Not Reproduce the Effect of Tlr2 Polymorphism at Position 82

Tlr2 can form dimers with several distinct co-ligands such as Tlr1 and Tlr6, the main co-receptors, Tlr10 and, possibly, Tlr2 itself, leading to differences in the pattern of molecules that each dimer can bind. A potential difference in the ability of Tlr2 isoforms to favor the dimerization with Tlr1 or Tlr6 may justify the differences between Tlr2^82ile^ and Tlr2^82met^ in their ability to regulate polarization of T cells. To address this point, we immunized SJL mice using as adjuvant IFA with Pam2CSK4, a selective ligand for Tlr2/Tlr6 dimers, or Pam3CSK4, a selective ligand for Tlr2/Tlr1 dimers, and studied the levels of mRNA specific for IL-17A, IL-13, IFN-γ, and FoxP3. Although LNC derived from mice challenged in the presence of Pam2CSK4 displayed a slightly higher production of IL-17A, the results showed no statistically significant difference between mice immunized using the two selective ligands (Figure 4B), thus suggesting that effects of polymorphism Ile82Met could hardly be explained by preferential dimerization of Tlr2 with either Tlr1 or Tlr6.

Notably, both Pam2CSK4 and Pam3CSK4 induced an antigen-driven-like upregulation of pro-inflammatory cytokines, but neither was able to induce antigen-driven upregulation of FoxP3 in LNC (Figure 4B). This observation indicates that Tlr2^82ile^ regulates the differentiation of Th cells toward antigen-dependent Treg phenotype after dimerization with co-receptor(s) other than Tlr1 or Tlr6, and excludes the possibility that Tlr2^82ile^ fails to block a default expansion of Treg cells.

### The Polymorphism Ile82Met Modifies the Binding Pockets of Tlr2

To have further indications about the mechanisms that sustain the ability of Met/Ile substitution at position 82 to modulate significantly the immune response, we examined *in silico* the effect of this polymorphism on the three-dimensional structure of Tlr2.

**Figure 5:**
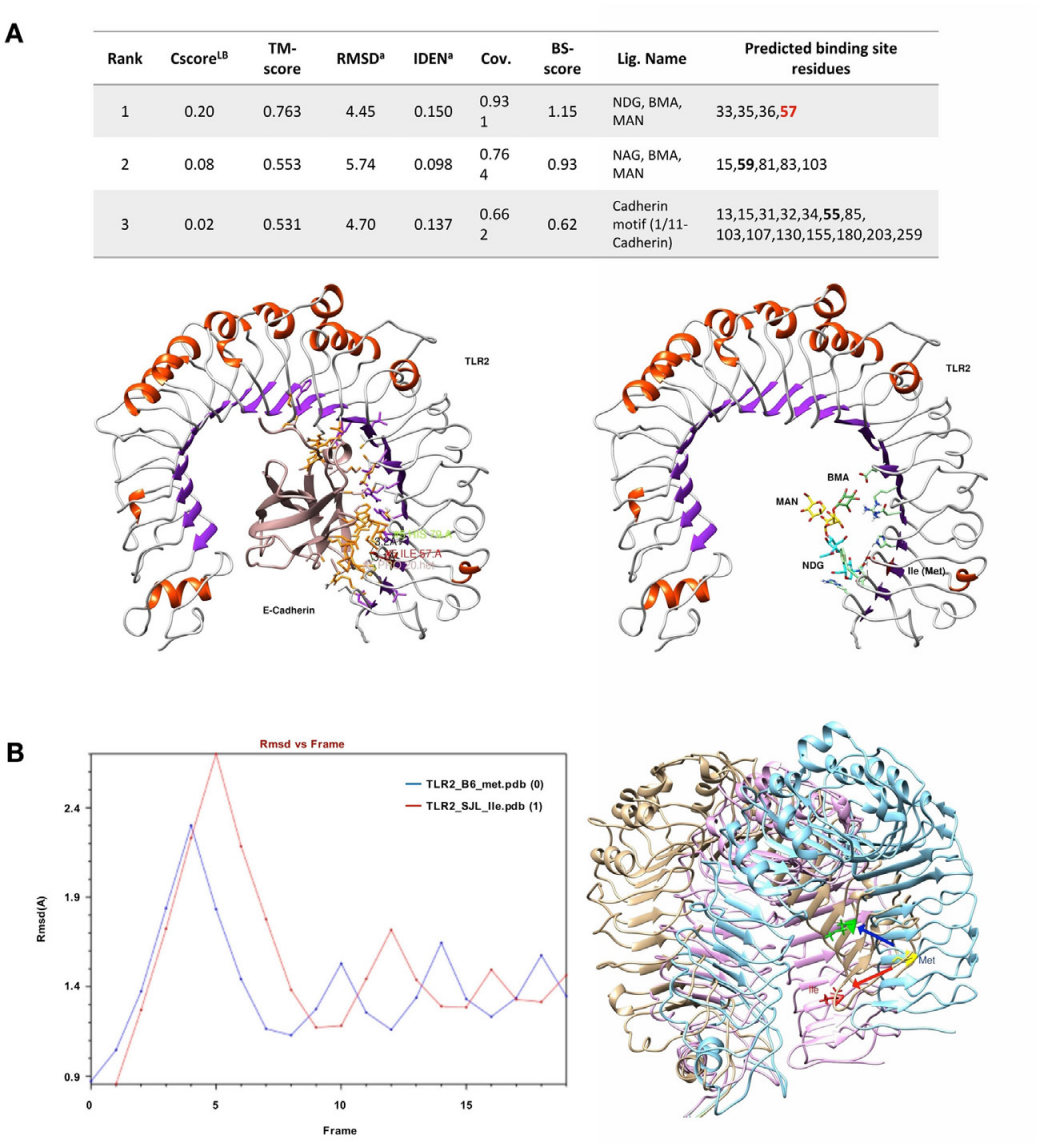

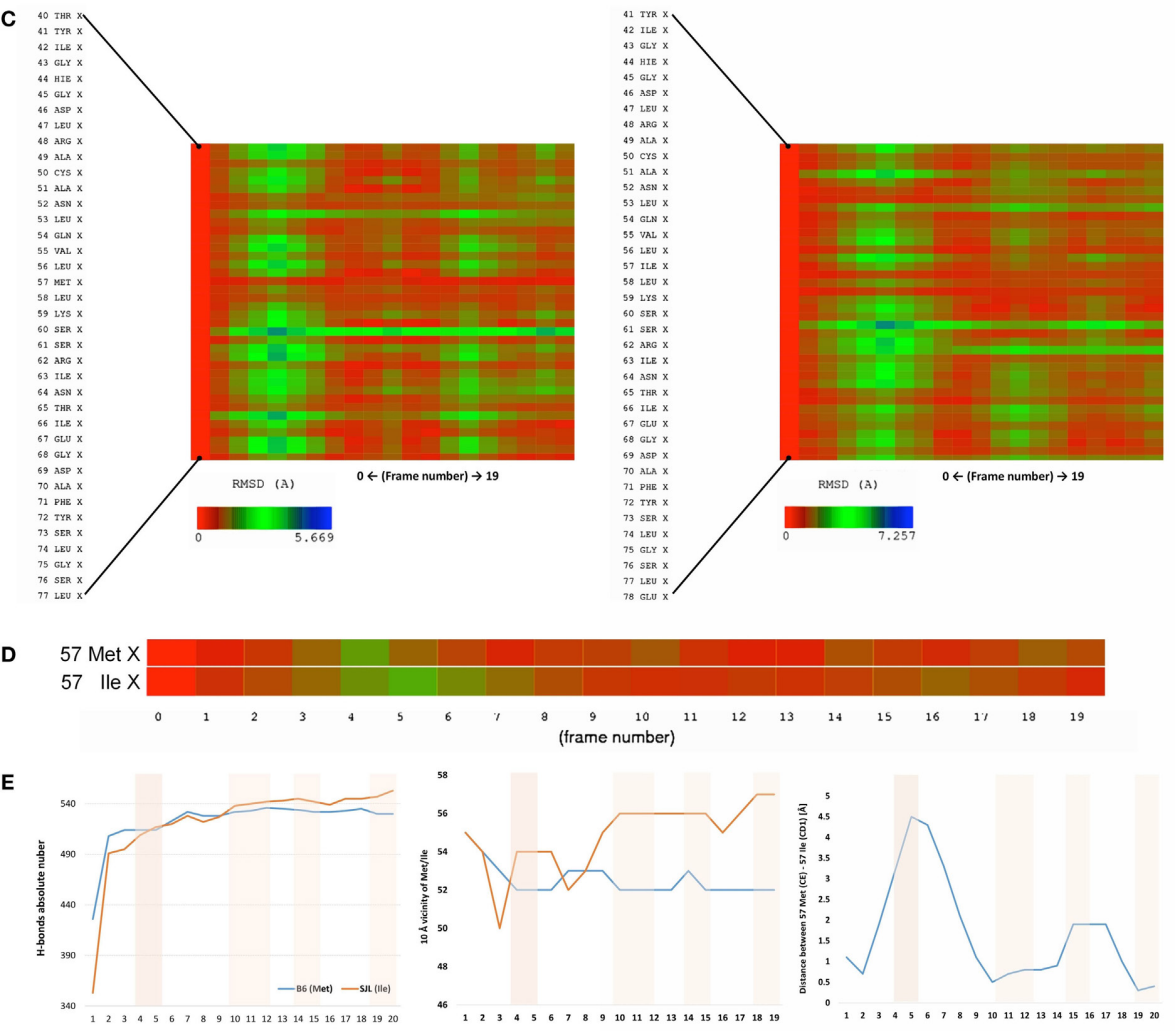
**Ligand-binding site prediction and molecular dynamics demonstrate 82Ile replacement of 82Met in Tlr2 to produce new sugar-binding sites and more rigid molecule**. **(A)** 82Ile SNP mutation increases likelihood of binding sugars such as NAG. Ligand-binding site prediction based on COFACTOR server reveal that multiple sugars such as NAG, BMA, MAN (left), and protein domains of cadherin family such as 1/11-cadherin (right). The binding interface residues are depicted in green and orange. The SNP mutation replacing 82Met with 82Ile in original Tlr2 sequence is direct binding site for NAG as shown in the table (below). The SNP is represented as Ile_57_ in the Tlr2 crystallography model (signal peptide is missing). **(B–E)** Molecular dynamics reveals 83Ile mutation causes less molecular fluctuations with greater amplitude and total and local intramolecular H-bonds inferring an increased molecular rigidity. **(B)** Tlr2 (82Met) and Tlr2 (82Ile) models were subject to energy minimization and solvation using AMBER MD suite (MDWeb server) and followed by normal mode analysis (anisotropic network model) (Protein Energy Landscape Exploration server). Trajectory analysis (RMSd vs. frames) revealed reduced number of peak fluctuations (left). Molecular displacement of the final energy minimization frame(s) of Tlr2 (Met) vs. Tlr2 (Ile), after C-terminus was held steady (right). **(C)** Zoom on time-dependent per-residue root mean square deviation plot of Tlr2 (Met) vs. Tlr2 (Ile) (left and right, respectively) with peak fluctuation maximum of 5.699 Å for Met82 vs. 7.275 Å for Ile82. In **(D)** only, Met vs. Ile RMSds by frame are shown. **(E)** Per-frame trajectory analysis of the number of established H-bonds in each of the Tlr2 variants (left). Number of H-bonds in 10 Å vicinity of the Met/Ile residues (pos 57) (middle). Per-frame distance between 57Met (CE) and 57Ile (CD1) of respective Tlr2 *wt* and *mutant* molecule structures (right).

The analysis of putative binding to variant Tlr2^82ile^ revealed that the Ile itself is a binding residue to several sugars, as determined by COFACTOR server, predicting ligand-binding sites using Tlr2 tertiary structure (Figure 5A). Cadherin preproprotein domain (aa 156–254), specifically cadherin 1/E-cadherin (*Homo sapiens*) and cadherin 11 (*Mus musculus*), was both found to bind to multiple residues. These included residue *79* and the mutated Met to Ile *82* (enumerated as residues *55* and *57* of the molecular model shown in Figure 5, *due to the absence of the leader peptide in the crystallography model*) (Figure 5A). Interestingly, the same amino acid sequence of E-cadherin predicted to bind to Tlr2 was found to bind also to *Listeria* invasion protein internalin, as found by blastp in pdb harbored sequences (not shown).

We modeled an 82Ile mutant Tlr2 version, using the crystallography model of Tlr2^82met^ and two different modeling approaches. First, one was using a template-based structure prediction modeling by I-Tasser web server. The second one was using the same Tlr2^82met^ model and rotamer substitution, followed by molecular dynamics (MD)-based molecular refinement. This MD refinement was achieved using energy minimization, salvation, and molecular equilibration (AMBER-based protocol, MDWeb server). MD simulation allowed for studying the atomic and molecular movement and interaction trajectories for a short fixed period of time, giving a view of the dynamical evolution of the system. We found that 82Ile inflicts lesser fluctuation of the energy minimized and solvated Tlr2 molecule in general, as shown by differing RMSd trajectory (RMSd vs. frames function, showing changes in molecular 3D structure in space) having less peaks in Tlr2^82ile^ vs. Tlr2^82met^ (Figure 5B). There was significant difference in their fluctuations and spatial shift in Ile holding β-sheet (visualized by Chimera trajectory analysis tool, after fixing the C-amino terminus of the Tlr2 chains and superimposing Tlr2^82met^ with Tlr2^82ile^ models).

Following RMSd fluctuations (global and Cα backbone, VMD Timeline tool) along the energy minimization trajectory, we found that maximal fluctuations differ within the range of a hydrogen bond, ~2 Å (from 5.669 to 7.257 Å) (Figure 5C). Even though residue 82Met–Ile was not the major residue displacement contributor within the whole molecule, it still demonstrated a center of fluctuation of more than ~1 Å (which is in the range of C–H or C–C chemical bond) (Figure 5D).

**Figure 6:**
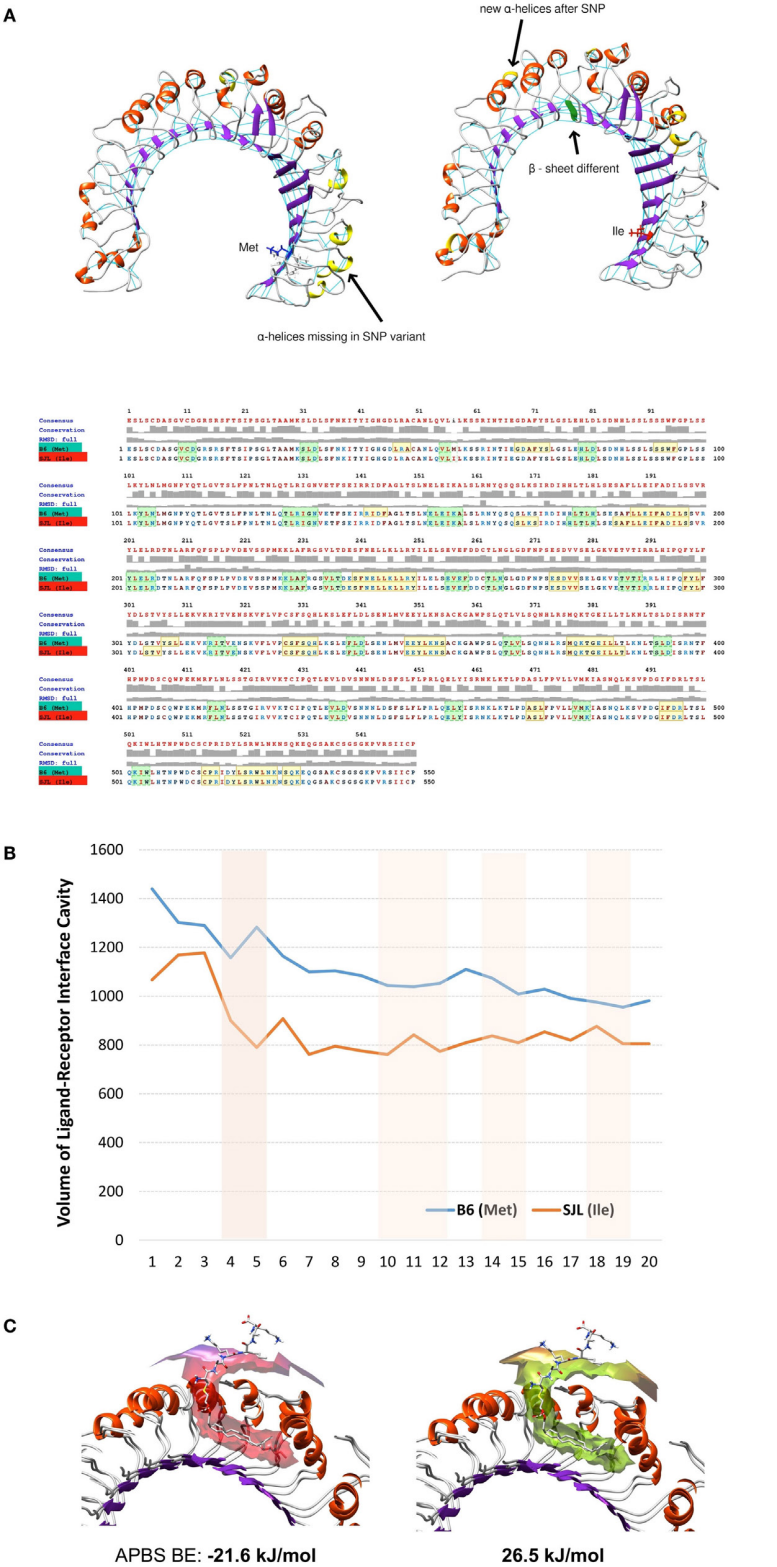
**Molecular dynamics and ligand–receptor energy assessment studies reveal 82Ile replacement of 82Met in Tlr2 to change molecules tertiary structure, ligand pocket volume, and binding energy, inferring lesser probability of ligand binding**. **(A)** Secondary structure differences revealed by molecular dynamics trajectory – structural models (top) and MUSCLE sequence alignment (bottom). Highlighted in yellow – α-helixes; in green – β-sheets. **(B)** Comparison of volume of ligand–receptor interface cavity measured using Intersurf in TLR2 *wt* (Met) vs. *mutated* (Ile) and the ligand PAM2CSK. The analysis is done on molecular dynamics trajectory. **(C)** Structural models of Tlr2 (Met)/(Ile) showing binding interface with PAM2CSK. APBS binding energies (BE) are shown below.

Following the total number of H-bonds formation dynamics in time, the dynamics in H-bonds number in a 10 Å vicinity of the 82Ile residue, and the distance between superimposed Tlr2^82met^ and Tlr2^82ile^ terminal C-atoms, we found that at specific time points the mutant Tlr2 had highest RMSd and residue displacement peaks, compared to wild-type (82Met) (as estimated by VMD Timeline tool: frames 4–5, 10–12, 14–15, and 19–20) (Figure 5E). This phenomenon corresponded to highest number of H-bonds in mutated Tlr2, suggesting that the moments when MD of Tlr2 caused its major deviations from wild-type structure, these very moments were strengthen with higher number of instant H-bonds. These fluctuation and displacement alterations were accompanied with changes in secondary structure of the Tlr2^82ile^ minimized energy form resulting in several α-helixes loss across the β-sheets at the side of 82Ile containing mutation (Figure 6A). Importantly, at same MD trajectory time points, the Tlr2^82ile^ model have also demonstrated decreased volume of its ligand–receptor interface cavity (Figure 6B). Further, the changes in the specific ligand-binding pocket itself were measured. It is usually smaller than the whole interaction interface. Pam2CSK4 pocket was estimated in *non-minimized* and *energy-minimized* conditions accordingly: 1440 and 981.5 Å^3^ (wild-type Tlr2^82met^); 1067 and 805.1 Å^3^ (mutated Tlr2^82ile^). Thus, in steady-state conditions, the ligand-binding pocket of the wild-type molecule was reduced by 458.5 Å^3^, while mutated molecule pocket was reduced by only 261.9 Å^3^. Overall, the mutation incurred 176 Å^3^ reduction in pocket volume. The data suggesting that the rigidity, and increased H-bonding ultimately resulted in decreased binding cavity and decreased likelihood of binding of cognate ligands to Tlr2 (Figure 6B). This reduced likelihood was validated by assessing the binding energy that was significantly increased, reflecting the observed molecular rigidity [ABPS-binding *E* of −21.6 kJ/mol (82Met) vs. 26.5 (82Ile) kJ/mol] (Figure 6C).

Overall, binding prediction and MD data suggested the mutant causes an increased molecular rigidity manifested by decreased number of higher amplitude molecular fluctuations, corresponding to an increased H-bonds formation. This was further supported by extension of three of the β-sheets, reduction of volume of ligand-binding cavity at the same time points as the ones with increased molecular rigidity, and an increased energy of ligand binding, resulting ultimately in lesser ligand-Tlr2^82ile^ receptor interactions.

### The Polymorphism on Position 82 of Tlr2 Modulates Severity of EAE

We have previously shown that the polymorphism of Tlr2 modulates T cell trafficking (30). Here, we have shown that each isoform of Tlr2 displayed a set of distinct pro- and anti-inflammatory properties with Tlr2^82ile^ promoting type 1/17 polarization and the expansion of antigen-specific FoxP3^+^ Tregs, and Tlr2^82met^ blocking the expansion of Tregs and that of production of IFN-γ and IL-17A. We therefore asked if the distinct pro- and anti-inflammatory effects of the two isoforms balanced each other, or if they impacted differently course and histopathology of an autoimmune disease model such as EAE.

**Figure 7:**
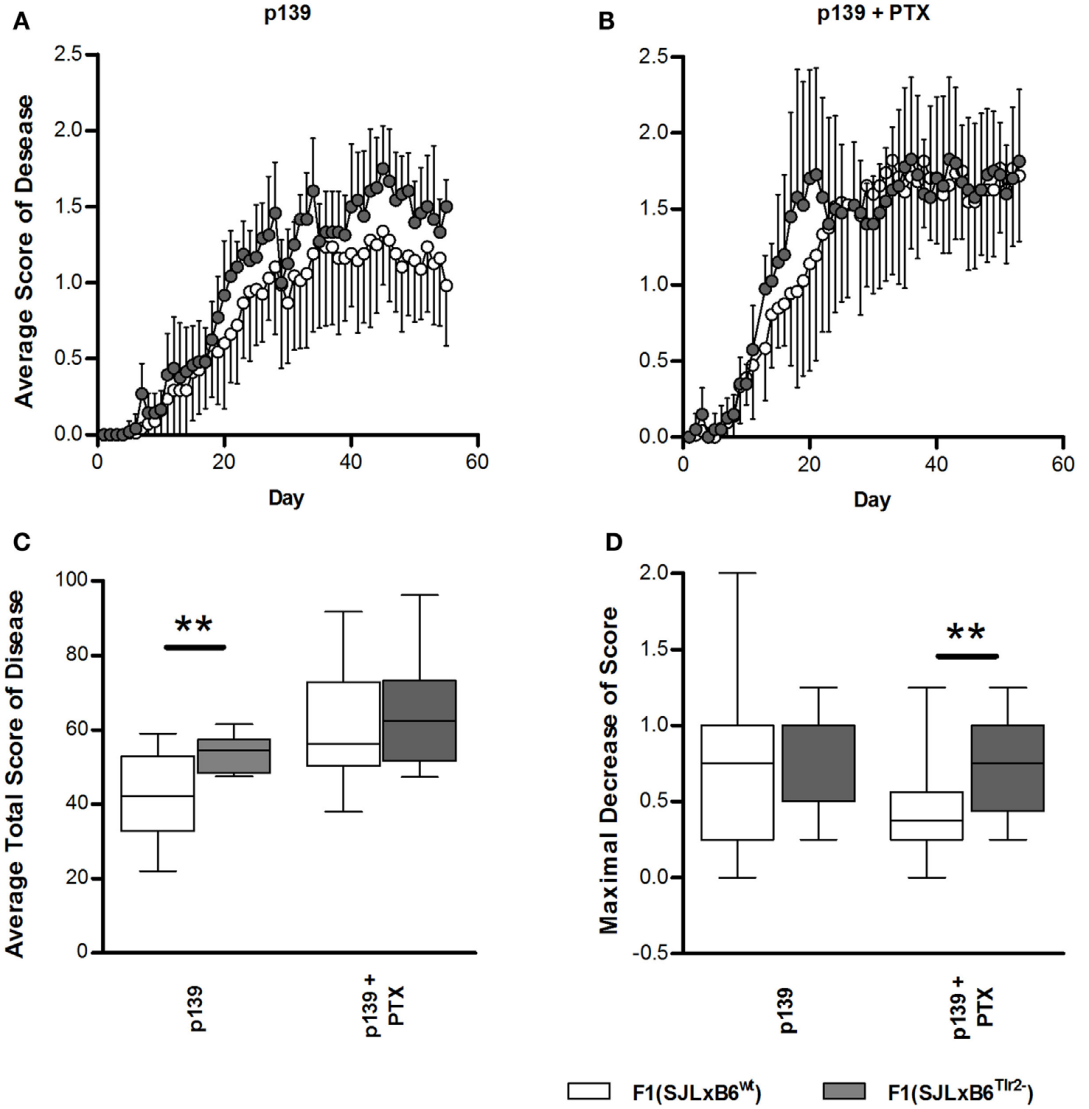
**Polymorphism at position 82 of Tlr2 modulates severity and course of EAE**. EAE score was evaluated as described in Section “Materials and Methods.” In all panels, data report the cumulated results of two independent experiments each comprising comparable numbers of mice from the genetic backgrounds examined in each panel; mice were evaluated daily for 60 days in a blind fashion with respect to genotype. Closed symbols and bars report data from F1 (SJL × B6^Tlr2−^) mice; the open symbols and bars report data from F1 (SJL × B6^wt^) mice. **(A)** The average disease score in 12 F1 (SJL × B6^Tlr2−^) and 17 F1 (SJL × B6^wt^) mice immunized with p139 without treatment with PTx. **(B)** The average disease score in 10 F1 (SJL × B6^Tlr2−^) and 18 F1 (SJL × B6^wt^) mice immunized with p139 and treated with PTx at day 0 and day 3 after immunization. **(C)** Total score of the diseases described in **(A,B)** are reported as boxplot. **(D)** Maximal decrease of disease score between day 20 and day 30 after immunization are reported as boxplot (two-tailed Mann–Whitney test, **p < 0.01).

To this aim, F1 mice were immunized s.c. with p139, and EAE development was monitored for the following 60 days (Figure 7). Overall, disease severity was milder than that previously reported for SJL mice; this effect may be due to the fact that F1 mice display a lower level of expression of the appropriate MHC restricting elements with respect to the parental strain. When we compared the two types of F1 mice, we found that F1 (SJL × B6^Tlr2−^) mice developed a slightly earlier and more severe disease than F1 (SJL × B6^wt^) mice (Figure 7A), indicating that the presence of Tlr2^82met^ attenuated EAE. PTx administration accelerates EAE in both F1 mice (Figure 7B), and the average total disease scores were increased in both types of F1 mice, more significantly in F1 (SJL × B6^wt^) mice (*p* = 0.0002 for F1 (SJL × B6^wt^) mice, *p* = 0.01 for F1 (SJL × B6^Tlr2−^) mice). However, after PTx administration, there was no statistical difference in the average total score of disease between the two groups, suggesting that PTx can overcome the difference in global severity due to Tlr2 (Figure 7C). Nevertheless, an interesting observation was that the clinical course of disease after treatment with PTx was not similar in F1 (SJL × B6^Tlr2−^) and F1 (SJL × B6^wt^) mice. In fact, a peak of disease severity was observed around day 20 after immunization in F1 (SJL × B6^Tlr2−^) mice, then a decrease of the average disease score between day 20 and day 30 followed by a new increase of the disease severity. The course of disease in F1 (SJL × B6^wt^) mice showed no signs of remission. The quantification of this difference following PTx administration is presented in Figure 7D showing the maximum of score decrease before day 30 after immunization, with F1 (SJL × B6^Tlr2−^) mice showing a significantly more considerable reduction of disease score compared to F1 (SJL × B6^wt^) mice (*p* = 0.02).

F1 (SJL × B6^Tlr2−^) mice have a reduced Tlr2 gene dosage, and we have previously demonstrated that the lower gene dosage of these mice did not lead to a difference of TLR2 expression (30). To assess if the reduction of the EAE severity was a function of the higher gene dosage in F1 (SJL × B6^wt^) with respect to F1 (SJL × B6^Tlr2−^) mice, we induced EAE in littermates that were either F1 (SJL × B6^Tlr2−^) mice, having one copy of functional Tlr2 of SJL origin, or F1 (SJL × B6^*ts*^), having two copies of functional Tlr2 of SJL origin. As described in Section “Materials and Methods,” B6^*ts*^ mice are mice of the B6 background carrying Tlr2 of SJL, and obtained by back-crossing of Tlr2 of SJL onto B6^Tlr2−^ for nine generations. No difference in the disease course and average total score was observed between these two groups (Figure S3 in Supplementary Material), confirming that it was the presence of the allele Tlr2^82met^ rather than gene dosage of Tlr2 that reduced the severity of EAE.

In conclusion, our data suggest that allele Tlr2^82met^ behaves as a dominant modulator of EAE in F1 (SJL × B6^wt^) mice, where it reduces the severity of EAE following challenge with p139 in adjuvant, in the absence of PTx administration. The presence of allele Tlr2^82ile^ alone, however, resulted in a higher degree of symptoms remission after PTx administration.

### Tlr2^82met^ Reduces Lesion Load in the Frontal Lobes

**Figure 8:**
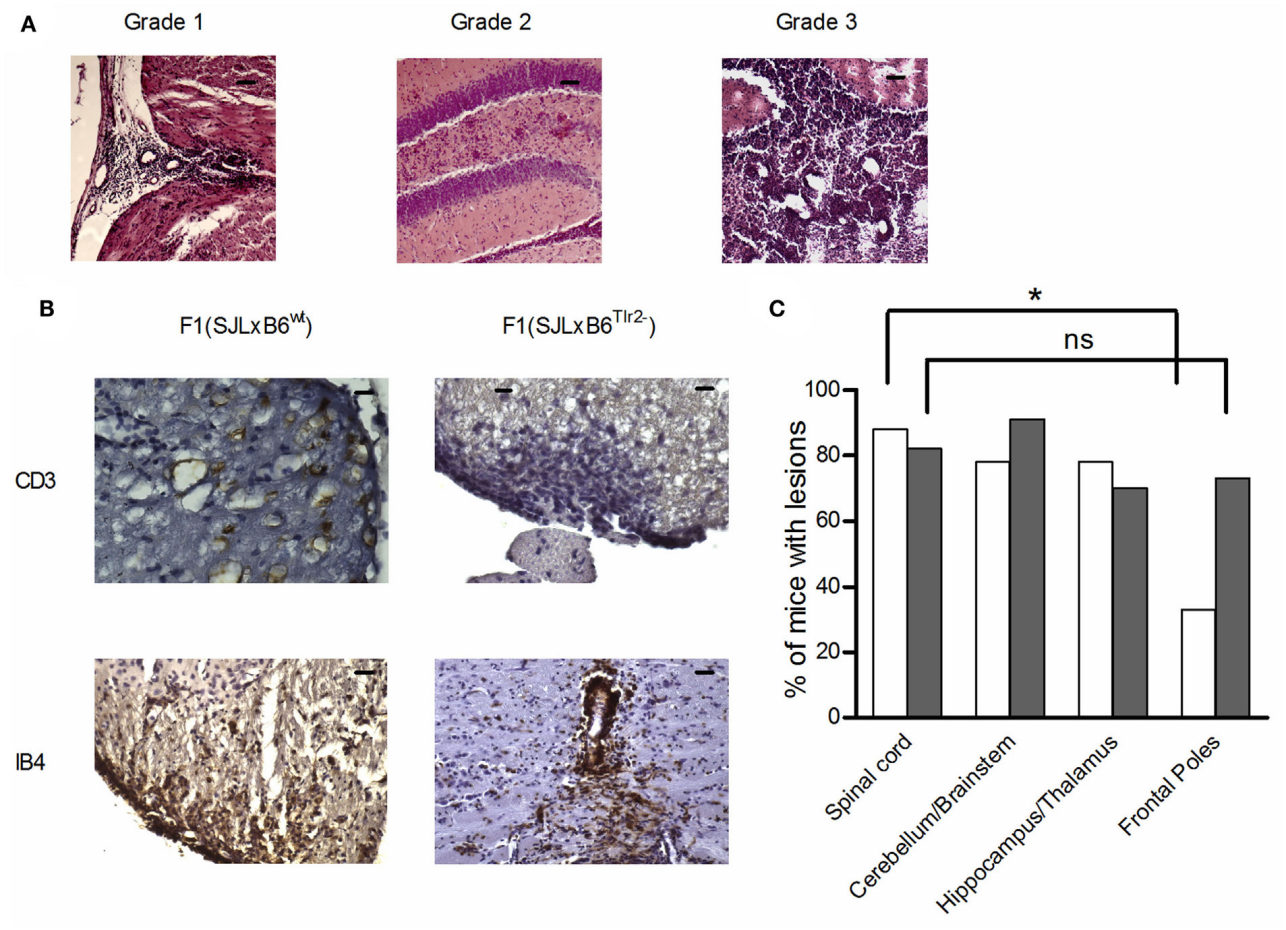

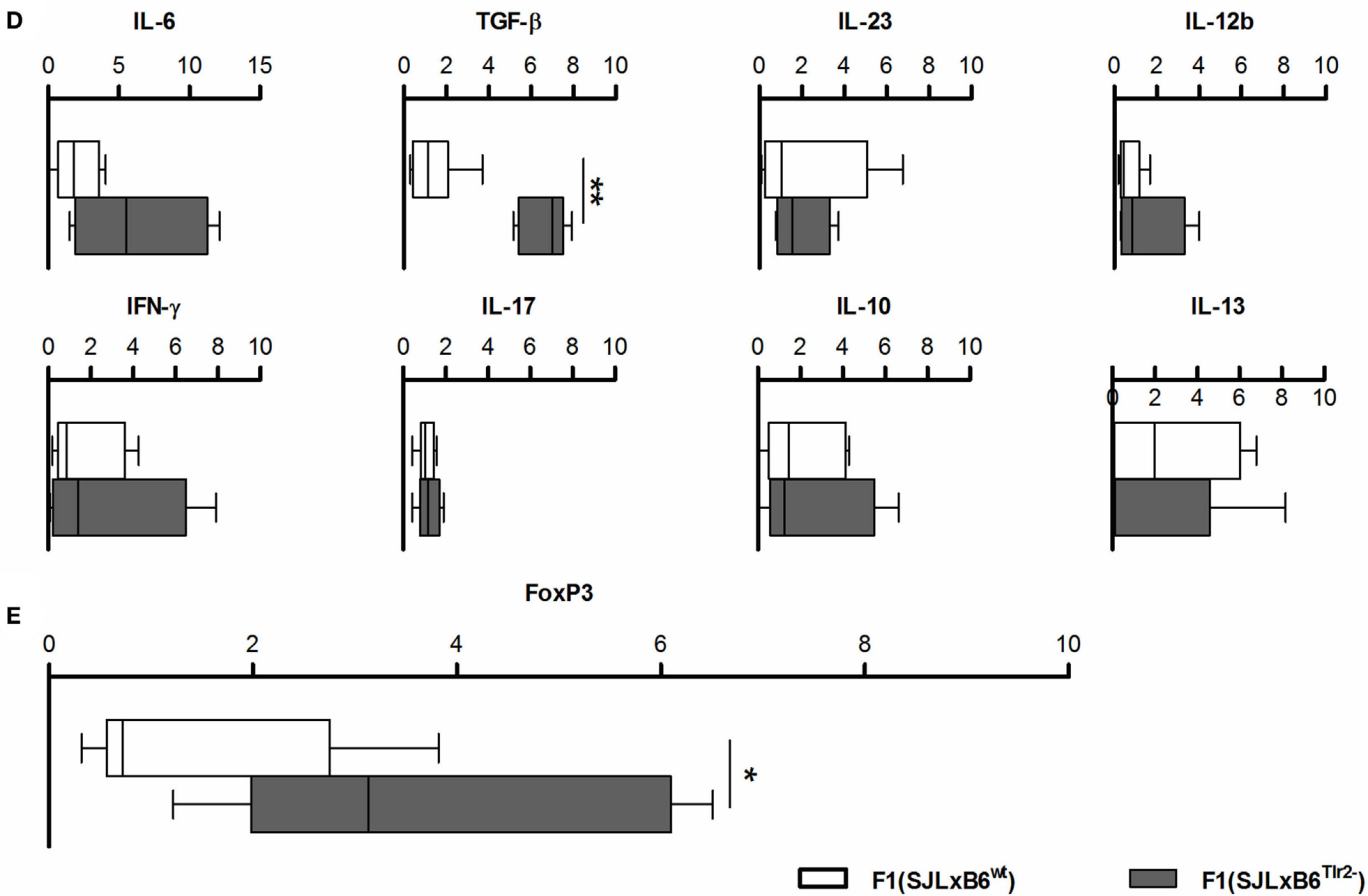
**Tlr2 genotype decreases the frequency of infiltrates in the frontal poles of the CNS and the cytokine and FoxP3 levels in CNS-infiltrating cells**. **(A–C)** 10 F1 (SJL × B6^wt^) (open bars) and 12 F1 (SJL × B6^Tlr2−^) (closed bars) mice were immunized s.c. with p139 in enriched CFA and treated with PTx. Data report the results of two independent experiments, each comprising mice of either genetic background. Mice were sacrificed at the disease peak and CNS histology was performed with H&E staining, as described in Section “Materials and Methods.” Part of these mice undergone IHC staining for CD3 and IB4. One section every 100 μm was examined, blind with respect to genotype and score of disease. **(A)** Representative images from H&E staining of mice brains coronal sections. Grade 1: presence of infiltrates limited to vessels and meninges (medulla oblongata); Grade 2: considerable presence of infiltrates in the parenchyma (hippocampus); and Grade 3: severe presence of infiltrates in the parenchyma (cerebellum), scale bar 100 μm. **(B)** Representative images from IHC staining for CD3 (scale bar 200 nm) and IB4 (scale bar 100 nm). **(C**) Frequencies of mice that present lesion distributions in different areas of the CNS in F1 (SJL × B6^wt^) (open bars) and F1 (SJL × B6^Tlr2−^) (closed bars) in at least one section per each of the area indicated (χ^2^ test, *p < 0.05). **(D,E)** Six F1 (SJL × B6^wt^) (open bars) and five F1 (SJL × B6^Tlr2−^) (closed bars) mice were immunized s.c. with p139 in enriched CFA. F1 (SJL × B6^Tlr2−^) mice were sacrificed when the score of disease had reached a value at least above 1. Each group of F1 mice was composed of three mice of two distinct litters. CNS-infiltrating cells were isolated by Percoll gradient and the levels of mRNA specific for **(D**) IFN-γ, IL-17, IL-10, IL-13, IL-12b, IL-23, TGF-β, and IL-6, and **(E**) FoxP3 were evaluated by qRT-PCR as described in Section “Materials and Methods.” The levels of mRNAs expression are expressed after normalization vs. the average value obtained from CNS-infiltrating cells of F1 (SJL × B6^wt^) mice. qRT-PCR reactions were performed in triplicate. Results are reported as boxplot (two-tailed Mann–Whitney test, *p < 0.05, **p < 0.01).

CNS lesions in EAE are largely limited to the spinal cord, a major difference with MS, in which lesions are largely present in the forebrain. Since we had previously observed that polymorphism Ile82Met of Tlr2 affects the trafficking properties of T cells, we next examined if it also influences the distribution of lesions in the CNS (Table 2; Figures 8A–C).

**Table 2:** **Presence and grade of infiltration in CNS of F1 (SJL × B6^wt^) and F1 (SJL × B6^Tlr2−^) during EAE**.

	F1 (SJL × C57^wt^)										F1 (SJL × C57^Tlr2−^)											
	A	B	C	D	E	F	G	H	J	*N* = 9	K	L	M	N	O	P	Q	R	S	T	U	*N* = 11
Score of disease	2.75	2.5	2	1.25	1.5	1.5	1.5	3	1.75		1.25	1.75	2.25	2.25	2	2	3	1.75	3	1.25	3	
Day of sacrifice	16	17	16	37	22	21	22	15	18		22	22	21	18	21	16	21	16	16	37	17	
										+(%)^b^												+(%)^b^
Frontal poles^a^	1	0	0	0	0	2	0	2	0	3 (33%)	1	1	0	1	1	1	0	1	1	0	1	8 (73%)
Hippocampus/thalamus^a^	1	0	2	0	1	1	1	1	1	7 (78%)	1	0	1	1	1	2	0	1	NA	0	2	7 (70%)
Cerebellum/brainstem^a^	2	0	3	0	1	1	1	1	1	7 (78%)	1	1	1	1	1	3	0	2	1	1	1	10 (91%)
Spinal cord^a^	2	3	1	2	1	0	1	2	1	8 (88%)	1	1	2	2	2	0	2	1	1	0	1	9 (82%)

*^a^For each CNS area, five regularly spaced serial sections were examined. Scores were attributed to each area according to the scale reported in Figure 7A. 0 = no infiltration found in any of the sections. Data report the highest score obtained*.

*^b^% of mice scoring ≥1 in at least one section from the indicated area/mice examine*.

We observed no qualitative differences in the neuropathological changes in the spinal cord, brain stem/cerebellum, and hippocampus/thalamus associated with Tlr2 polymorphism (Figure 8C). Notably, 73% of F1 (SJL × B6^Tlr2−^) mice showed the presence of lesions in the frontal lobes, whereas only 33% of F1 (SJL × B6^wt^) mice exhibited lesions in these areas. We observed a statistically significant lower lesion load between the frontal lobes and spinal cord only in the F1 (SJL × B6^wt^) mice (*p* = 0.049), suggesting that Tlr2^82ile^ promotes and Tlr2^82met^ prevents the infiltration of the frontal lobes.

### Cytokine and FoxP3 Profile in the CNS

As shown in Figures 8A,B, the neuropathology features of the lesions in both F1 mice appear similar, including the presence of CD3^+^ cells and activated microglia (Figure 8B). When we assessed the profile of mRNAs specific for cytokine in cells infiltrating the CNS at the peak of disease, we found a statistically significant difference for TGF-β production, which was significantly higher in the CNS of F1 (SJL × B6^Tlr2−^) mice (Figure 8D). No difference was observed in the amount of IL-12, IFN-γ, IL-17A, and IL-23 mRNAs. Consistent with the data obtained in the LNC (Figure 2), the cells infiltrating the CNS of F1 (SJL × B6^Tlr2−^) mice displayed also significantly higher levels of FoxP3-specific mRNA (Figure 8E).

## Discussion

In the present work, we exploited the polymorphism Ile82Met to investigate the role of Tlr2 in the proinflammatory and anti-inflammatory effects of adjuvant containing Mtb, *in vivo*. We found that each isoform of Tlr2 displayed a set of distinct pro- and anti-inflammatory properties. Upon usage of the same adjuvant, Tlr2^82ile^ promotes polarization of T cell mediated responses toward a type 1/17 phenotype and the expansion of antigen-specific FoxP3^+^ Tregs, whereas Tlr2^82met^ blocks the expansion of Tregs and reduces the production of IFN-γ and IL-17A. Both pro- and anti-inflammatory effects of the polymorphisms appear associated with modification of the secretion of the directive cytokines produced by the APC, and we found no evidence of a role for Tlr2/Tlr1 or Tlr2/Tlr6 dimers in the regulation of expression of FoxP3 by CD4^+^CD25^+^ T cells, *in vitro* directly on T cells, and *in vivo*. When we studied *in silico* the effect of polymorphism at position 82 on Tlr2, we observed that this residue influences at a distance the primary binding pocket of Tlr2, and also is directly involved in a candidate secondary pocket that may bind sugars and cadherins. We also found significant differences in the EAE course and neuropathology in F1 (SJL × B6^wt^) vs. F1 (SJL × B6^Tlr2−^) mice, indicating that effects of Tlr2 polymorphism on the immune response are translated into immunopathology.

Thus, Tlr2 mediates *in vivo* pro- and anti-inflammatory effects of Mtb, and can modify the clinical course and neuropathology during EAE.

Thus, the presence of functional differences between the polymorphic forms of Tlr2 provided a unique opportunity to dissect its role *in vivo*, bypassing the limitation of KO models in which knocking-out Tlr2 abolishes all of the distinct effects of its various dimers. Although, according to the limited genetic differences that exist between the two types of F1 mice, the absolute values of differences are small, for several of them statistic significance is achieved. Thus, our observations define Tlr2 as a molecular link between infectious history and modulation of the course of autoimmunity, in which, however, it plays a janus role.

Tlr2 promotes the secretion of directive cytokines, such as IL-12 and IL-23 by DCs (53, 54) and acts directly on T cells, in particular regulating in the periphery the balance between Th17 and Tregs (24, 25). It has been reported that T cells migrated at the sites of autoimmune inflammation have a Th1/Th17 cell-like phenotype, producing high levels of IL-17 and IFN-γ (55, 56). Even if the mechanisms that govern plasticity of Th17 cells are unclear, the transcription factor RORγ-T does not appear to be involved in the development of T cells expressing this hybrid Th1/Th17 profile (57). TLR2 has been shown to convert FoxP3^+^ Treg to Th17 (25), and to suppress polarization of Th cells to a Treg phenotype. In turn, Th17 cells can transdifferentiate to Treg during the resolution of inflammation driven by anti-CD3 administration (58).

Our finding that Tlr2^82met^ determines a decrease in the amount of IFN-γ, IL-17A, and of the mRNA specific for T-bet, following *in vivo* challenge and after p139 stimulation *in vitro*, but not for RORγ-T is consistent with the hypothesis that Tlr2^82ile^ promotes the acquisition of a Th1/Th17 cell-like phenotype whose development is linked to the expression of T-bet, but does not require expression of RORγ-T.

In tandem, Tlr2^82ile^ promoted the acquisition of an antigen-dependent Treg phenotype, detected *in vitro* (Figures 2 and 3), and suggested *in vivo* in the CNS by mRNA evaluation (Figure 8).

It has been reported that Treg development is regulated by IL-1R through MyD88 (59). It has also been reported that IL-1b promotes Th17 polarization of naive T cells by favoring the alternative splicing of FoxP3-specific mRNA thus generating an isoform of FoxP3 lacking exon 7 that promotes the secretion of IL-17 (60). The findings reported here point to Tlr2 as a new player in the MyD88-dependent modulation of Treg phenotype.

It will be important to establish if the T cells able to transdifferentiate between Th1/17 and Treg represent a defined population regulated by Tlr2, or rather if Tlr2 regulates independently the acquisition of a type 1/17 and of a Treg phenotype. In our previous work, we showed that, in addition to the expected robust CD4-mediated IFN-γ response (61), challenge of SJL mice with p139 in CFA elicits also a smaller, but measurable, p139-specific CD8-mediated response (4). It has been reported that induction of Tc17 phenotype by CD8^+^ cells following fungal infections is dependent on Tlr2 and MyD88, while induction of Tc1 phenotype is not (62). Thus, we cannot formally exclude that a part of the effect of the Tlr2 haplotype on the polarization of the response to type1/17 can also involve p139-specific CD8 T cells. However, in our model, both IFN-γ and IL-17A are regulated in parallel by Tlr2 haplotype, thereby hinting that we may rather be dealing with a CD4-related effect.

Tlr2 has the widest repertoire of ligands among pathogens’ receptors, co-acting with a variety of other molecules. Its main site binding for peptidoglycan lies distant from the polymorphic residue 82 (11, 63). We showed that the functional differences between Tlr2^82ile^ and Tlr2^82met^ could not be explained by the preferential dimerization with Tlr1 or Tlr6 (Figure 4), hinting that other co-receptors and ligand(s) are most likely responsible for the induction of antigen-specific FoxP3^+^ T cells. To gain some hints about how a substitution at this position can affect the functions of TLR2, we examined *in silico* the structural consequences of this polymorphism. The binding prediction, but especially MD modeling and ligand-binding energy assessment revealed that 82Ile replacement of 82Met created changes favoring new small molecules (sugars and E-cadherin) binding and perturbation in normal molecular fluctuations. The latter is based on tertiary structure modification due to loss of α-helical and increase of β-sheet structural properties of Tlr2. These changes ultimately result in more rigid molecular fluctuations, decreased ligand–receptor pocket volume, and non-favorable energy of binding, stabilized by an increased number of H-bonds. Overall, MD methodology is superior to mere ligand–receptor docking studies, providing an insight on molecular specificities causing structural–functional relationships. In this study, dynamics and energy changes incurred by 82Met mutation ultimately resulted in unfavorable binding energy and lesser probability of further signal transduction.

The results indicate two possible mechanisms, which are not mutually exclusive. The first suggests that the position 82 influences the flexibility and size of the main binding grove, with 82Met resulting in a larger and more flexible pocket; as a consequence, the binding pocket of Tlr2^82met^ may accommodate a wider repertoire of ligands, thus explaining its dominance in heterozygous F1. In addition, we identified a surface around residue 82, potentially able to bind sugars or (self)-proteins, whose binding properties were modified by the substitution. This latter observation raises the issue of the possibility that large repetitive molecules may bind to Tlr2 along a model of interaction similar to that of the other Tlrs, i.e., involving its horseshoe domain.

To the best of our knowledge, there are no common polymorphisms of Tlr2 in any of the first three LRR regions of the molecule in humans (64). We have recently shown that PE-PGRS33 of Mtb enhances the entry of Mtb into the macrophages through Tlr2 (65), and preliminary data indicate that residue 82 may be directly involved in the interaction between PE-PGRS33 and Tlr2. An evolutionary pressure exerted by Mtb may be a reason for the lack of polymorphisms in this region, in humans.

Data on the role of Tlr2 in EAE and MS are conflicting. It was reported that Tlr2-KO mice develop EAE (66), but evidences also exist indicating that Tlr2 plays a role in sustaining EAE (67). Tlr2 ligands were found in the CNS of MS patients (68), and Tlr2 is expressed on several cell types involved in disease determination (25). The possibility that activation of Tlr2 influences the course of MS is still debated (69).

Treatment with isoniazid (INH) still represents the centerpiece for treatment of tuberculosis. INH is frequently associated with the development of drug-induced LES. Although it was originally supposed that INH acts by haptenization of self-proteins, more recent data suggest that it alters the control mechanisms of immune responses (70). The case of a 39-year-old man that developed a primary progressive MS after therapy with INH to treat a latent Tbc (71) supports the hypothesis that latent Mtb infections can actually provide anti-inflammatory effects in some cases.

As mentioned above, both Tlr2^82met^ and Tlr2^82ile^ have anti-inflammatory properties that, however, impact differently on the clinical course of EAE. Tlr2^82met^ leads to a relative reduction of the production of proinflammatory cytokines and a milder disease severity. In F1 mice expressing only Tlr2^82ile^, we observed a relatively higher level of proinflammatory cytokines but also the significant presence of antigen-specific CD4^+^CD25^+^FoxP3^+^ T cells and found the presence of increased levels of FoxP3 mRNA in the CNS at the peak of disease, associated with a small but significant remission of symptoms. The depletion of Treg cells in SJL mice was shown to increase EAE severity and to reduce late remission (72). Our findings are consistent with the hypothesis that inducible Tregs may have a role in the induction of remission rather than in regulating the overall severity of the disease.

The distribution of inflammatory infiltrates in the CNS is one of the main differences between MS and most models of EAE (73), and the distribution of lesions in the brain of MS patients is extremely variable, contributing to the poor predictability of the clinical disease. Our data showed that also the presence of mild infiltrates in the frontal poles is dictated by the Tlr2 haplotype (Figure 8). Trafficking of T cells is correlated with Th polarization (74), which we show to be modulated by Tlr2 haplotype. In addition, we report that CCR2 mRNA is likely directly regulated by Tlr2 haplotype (Table 1). CCR2 has a central role in the transmigration of blood cells into the CNS (75). We have also found some mild differences of the expression of cell surface markers related to trafficking across endothelia (namely, CD49 and CD44) apparently linked to Tlr2 haplotype in antigen-activated T cells (manuscript in preparation). To our knowledge, there are no reports of trafficking pathways involved in the recruitment of self-reactive T cells to specific areas of the CNS. Thus, the possibility of the involvement of these molecules in the selection of areas of the CNS as targets of inflammation needs to be assessed.

The finding that the odorant receptors (ORs) represent one of the pathway regulated by Tlr2 polymorphism in our microarray analysis was unexpected. OR genes comprise more than 1200 genes in mouse. Two groups reported a functional relationship between ORs and leukocytes, showing that ORs are involved in the maintenance of blood leukocytes precursors in *Drosophila* (76) and that ORs and taste receptors distribute differentially among the subpopulation of leukocytes (77). A cluster of ORs is located near to the MHC locus, and it was suggested to link mating behavior to immune responses (78), although this hypothesis has not been confirmed (79). However, none of the ORs, which we found to be regulated by Tlr2, belong to this cluster, while they are spread among various chromosomes. Toll-6 and Toll-7 in *Drosophila* have been shown to be involved in the regulation of the olfactory circuits by neurons (80). Whether the findings reported here indicate the preservation of an essential pathway, it requires further investigation.

Our study point to Tlr2 as a key molecule in the pathway mediating the janus effects of administration of mycobacterium-derived products to self- and allo-reactive immune responses *in vivo*. Given the complex and in contradictory effects that PRRs and in particular Tlr2 engagement has on immune responses, a better definition of their role in human disease is needed, before interference with these molecules can be proposed as a safe therapeutic approach.

## Author Contributions

FR, AP, GM, and GS conceived and designed the experiments. AP, GM, GS, MV, AP, MCG, and MC performed the experiments. FR, AP, GM, GS, GC, SH, MF, GD, and AS analyzed the data. MC, AS, GD, GC, SH, and MF contributed reagents/materials/analysis tools. FR wrote the paper.

## Conflict of Interest Statement

The authors declare that the research was conducted in the absence of any commercial or financial relationships that could be construed as a potential conflict of interest.

## Footnotes

^1^http://zhanglab.ccmb.med.umich.edu/COFACTOR/

^2^http://mmb.irbbarcelona.org/MDWeb/

^3^https://pele.bsc.es

^4^http://www.ks.uiuc.edu/Research/vmd/
