# Supplementary material for: Toll-Like Receptor 2 Mediates In Vivo Pro- and Anti-inflammatory Effects of Mycobacterium Tuberculosis and Modulates Autoimmune Encephalomyelitis
Source: Front Immunol. 2016 May 24;7:191. doi: 10.3389/fimmu.2016.00191 (PMC4878199; doi:10.3389/fimmu.2016.00191)
Supplement: Supplementary file 1 [file Table_1.DOCX]

**Supplementary Table 1a. List of DEGs selected with Fold Change >1.5 (the most up-regulated). F1(SJLxB6^Tlr2-^) vs. F1(SJLxB6^wt^)**

| **Gene Symbol** | **Fold Change (linear)** | **ANOVA p-value** | **GO Biological Process Term** |
| --- | --- | --- | --- |
| **Olfr1417** | 2,9 | 0,03273 | signal transduction; G-protein coupled receptor signaling pathway; sensory perception of smell; response to stimulus; detection of chemical stimulus involved in sensory perception of smell |
| **Olfr380** | 2,49 | 0,070951 | signal transduction; G-protein coupled receptor signaling pathway; sensory perception of smell; response to stimulus; detection of chemical stimulus involved in sensory perception of smell |
| **Tubb3** | 2,49 | 0,098411 | GTP catabolic process; microtubule-based process; mitotic nuclear division; axon guidance; neuron differentiation; protein polymerization |
| **Il17f** | 2,42 | 0,462485 | proteoglycan metabolic process; inflammatory response; negative regulation of angiogenesis; regulation of transforming growth factor beta receptor signaling pathway; cytokine biosynthetic process; lymphotoxin A biosynthetic process; regulation of interleukin-2 biosynthetic process; regulation of interleukin-6 biosynthetic process; regulation of interleukin-8 biosynthetic process; regulation of granulocyte macrophage colony-stimulating factor biosynthetic process; positive regulation of transcription from RNA polymerase II promoter; cartilage development; positive regulation of cytokine production involved in inflammatory response |
| **Olfr1388** | 2,35 | 0,007553 | signal transduction; G-protein coupled receptor signaling pathway; sensory perception of smell; detection of chemical stimulus involved in sensory perception of smell |
| **Rorc** | 2,27 | 0,182996 |  |
| **Il22; Iltifb** | 2,07 | 0,449359 | regulation of tyrosine phosphorylation of Stat3 protein; positive regulation of transcription from RNA polymerase II promoter; negative regulation of inflammatory response; reactive oxygen species metabolic process |
| **Olfr305** | 2,05 | 0,049758 | signal transduction; G-protein coupled receptor signaling pathway; sensory perception of smell; response to stimulus; detection of chemical stimulus involved in sensory perception of smell |
| **Il23r** | 1,97 | 0,176079 | positive regulation of defense response to virus by host; immune system process; leukocyte mediated immunity; positive regulation of T-helper 1 type immune response; inflammatory response; JAK-STAT cascade; cytokine-mediated signaling pathway; response to lipopolysaccharide; negative regulation of interleukin-10 production; positive regulation of interferon-gamma production; positive regulation of interleukin-12 production; response to interferon-gamma; interleukin-23-mediated signaling pathway; innate immune response |
| **Il1r1** | 1,87 | 0,25352 | signal transduction; cytokine-mediated signaling pathway; regulation of inflammatory response; interleukin-1-mediated signaling pathway; response to interleukin-1 |
| **Olfr59; Olfr409-ps1** | 1,84 | 0,170308 | signal transduction; G-protein coupled receptor signaling pathway; sensory perception of smell; response to stimulus; detection of chemical stimulus involved in sensory perception of smell |
| **Olfr820** | 1,83 | 0,062692 | signal transduction; G-protein coupled receptor signaling pathway; sensory perception of smell; response to stimulus; detection of chemical stimulus involved in sensory perception of smell |
| **Olfr1196; Olfr1197** | 1,76 | 0,432315 | signal transduction; G-protein coupled receptor signaling pathway; sensory perception of smell; response to stimulus; detection of chemical stimulus involved in sensory perception of smell |
| **Il17a; Mir133b** | 1,68 | 0,755016 | inflammatory response; positive regulation of interleukin-23 production; positive regulation of osteoclast differentiation; positive regulation of transcription from RNA polymerase II promoter; cellular response to interleukin-1; cellular response to glucocorticoid stimulus; fibroblast activation; positive regulation of cytokine production involved in inflammatory response; positive regulation of gene expression; negative regulation of neuron maturation; response to oxygen levels |
| **Olfr488** | 1,61 | 0,233521 | signal transduction; G-protein coupled receptor signaling pathway; sensory perception of smell; response to stimulus; detection of chemical stimulus involved in sensory perception of smell |
| **Olfr1217** | 1,59 | 0,095087 | signal transduction; G-protein coupled receptor signaling pathway; sensory perception of smell; response to stimulus; detection of chemical stimulus involved in sensory perception of smell |
| **Olfr1056** | 1,59 | 0,116859 | signal transduction; G-protein coupled receptor signaling pathway; sensory perception of smell; response to stimulus; detection of chemical stimulus involved in sensory perception of smell |
| **Olfr582** | 1,58 | 0,070125 | signal transduction; G-protein coupled receptor signaling pathway; sensory perception of smell; response to stimulus; detection of chemical stimulus involved in sensory perception of smell |
| **Olfr1023** | 1,58 | 0,161614 | signal transduction; G-protein coupled receptor signaling pathway; sensory perception of smell; response to stimulus; detection of chemical stimulus involved in sensory perception of smell |

**Supplementary Table 1b. List of DEGs selected with Fold Change <-1.5 (the most downp-regulated). F1(SJLxB6^Tlr2-^) vs. F1(SJLxB6^wt^)**

| **Gene Symbol** | **Fold Change (linear) (KO vs. WT)** | **ANOVA p-value (KO vs. WT)** | **GO Biological Process Term** |
| --- | --- | --- | --- |
| **Cpa3** | -5,21 | 0,002403 | regulation of angiotensin levels in blood; proteolysis |
| **Iglv1** | -5,09 | 0,048346 | immune system process; antigen processing and presentation of peptide antigen via MHC class I |
| **Olfr441; Olfr237-ps1** | -3,84 | 0,403174 | signal transduction; G-protein coupled receptor signaling pathway; sensory perception of smell; response to stimulus; detection of chemical stimulus involved in sensory perception of smell |
| **LOC102641542; Iglc2; Iglv3; Iglv1; 2010309G21Rik** | -3,65 | 0,178342 | immune system process; antigen processing and presentation of peptide antigen via MHC class I |
| **Cd79a; LOC102634693** | -2,85 | 0,013779 | immune system process; cell surface receptor signaling pathway; B cell differentiation; B cell proliferation; B cell activation; B cell receptor signaling pathway |
| **Scd1** | -2,71 | 0,003629 | lipid metabolic process; fatty acid metabolic process; fatty acid biosynthetic process; positive regulation of cholesterol esterification; cholesterol esterification; negative regulation of growth of symbiont in host; defense response to Gram-positive bacterium; white fat cell differentiation.. |
| **Olfr1290; Olfr1292-ps1** | -2,25 | 0,044207 | signal transduction; G-protein coupled receptor signaling pathway; sensory perception of smell; response to stimulus; detection of chemical stimulus involved in sensory perception of smell |
| **Ctla2a; Ctla2b** | -2,2 | 0,000256 | negative regulation of protein processing; regulation of regulatory T cell differentiation; negative regulation of inflammatory response; proteolysis; negative regulation of endopeptidase activity |
| **Ly6d** | -2,2 | 0,016784 | lymphocyte differentiation; response to stilbenoid |
| **Gzmm; Cdc34** | -2,16 | 0,004002 | proteolysis; cytolysis; protein polyubiquitination; cell cycle; protein ubiquitination; proteasome-mediated ubiquitin-dependent protein catabolic process; positive regulation of neuron apoptotic process.. |
| **Cd19** | -2,12 | 0,015365 | B cell receptor signaling pathway |
| **Ebf1** | -2,1 | 0,001761 | transcription, DNA-templated; regulation of transcription, DNA-templated; multicellular organismal development; positive regulation of transcription, DNA-templated |
| **Mzb1; Prob1** | -2,09 | 0,000977 | positive regulation of immunoglobulin biosynthetic process; positive regulation of cell proliferation; regulation of B cell proliferation; integrin activation; regulation of cell proliferation; negative regulation of glucose import in response to insulin stimulus; biological_process |
| **Il5** | -2,08 | 0,05336 | immune response; positive regulation of cell proliferation; cytokine-mediated signaling pathway; positive regulation of B cell proliferation; positive regulation of eosinophil differentiation; positive regulation of transcription, DNA-templated; positive regulation of JAK-STAT cascade; positive regulation of immunoglobulin secretion; inflammatory response |
| **Cr2** | -2,07 | 0,003532 | immune system process; complement receptor mediated signaling pathway; complement activation, classical pathway; B cell differentiation; B cell proliferation; B cell activation; innate immune response |
| **Cd86** | -2 | 0,003684 | toll-like receptor signaling pathway; immune system process; negative regulation of T cell anergy; positive regulation of T cell proliferation; positive regulation of activated T cell proliferation; B cell activation; defense response to virus; response to yeast; T cell proliferation involved in immune response; aging; response to lipopolysaccharide; response to interferon-gamma; myeloid dendritic cell differentiation.. |
| **H2-DMb2; H2-DMb1** | -1,91 | 0,002244 | immune system process; antigen processing and presentation of peptide or polysaccharide antigen via MHC class II; antigen processing and presentation; antigen processing and presentation of exogenous peptide antigen via MHC class II; chaperone mediated protein folding requiring cofactor; immune response; antigen processing and presentation of peptide antigen via MHC class I |
| **Il10** | -1,89 | 0,042872 |  |
| **Olfr1163** | -1,84 | 0,048988 | signal transduction; G-protein coupled receptor signaling pathway; sensory perception of smell; response to stimulus; detection of chemical stimulus involved in sensory perception of smell |
| **Socs3** | -1,83 | 0,014557 | regulation of protein phosphorylation; signal transduction; JAK-STAT cascade; negative regulation of signal transduction; intracellular signal transduction; regulation of growth; negative regulation of apoptotic process; regulation of cell differentiation; positive regulation of cell differentiation; negative regulation of insulin receptor signaling pathway.. |
| **Cd180** | -1,83 | 0,004949 | B cell proliferation involved in immune response; immune system process; inflammatory response; positive regulation of lipopolysaccharide-mediated signaling pathway; innate immune response; cellular response to lipopolysaccharide |
| **Tnfrsf23** | -1,83 | 0,006927 | negative regulation of extrinsic apoptotic signaling pathway via death domain receptors |
| **Olfr889** | -1,82 | 0,019916 | signal transduction; G-protein coupled receptor signaling pathway; sensory perception of smell; response to stimulus; detection of chemical stimulus involved in sensory perception of smell |
| **Cd72** | -1,8 | 0,007364 |  |
| **Mllt11** | -1,79 | 0,011772 | positive regulation of apoptotic process; positive regulation of transcription, DNA-templated; positive regulation of mitochondrial depolarization; positive regulation of release of cytochrome c from mitochondria; apoptotic signaling pathway; extrinsic apoptotic signaling pathway; intrinsic apoptotic signaling pathway |
| **Blnk** | -1,79 | 0,00971 | positive regulation of signal transduction; intracellular signal transduction; B cell activation |
| **Olfr1128; Gm13740** | -1,77 | 0,035837 | signal transduction; G-protein coupled receptor signaling pathway; sensory perception of smell; response to stimulus; detection of chemical stimulus involved in sensory perception of smell |
| **Cd74** | -1,74 | 0,002626 |  |
| **Olfr584** | -1,71 | 0,049419 | signal transduction; G-protein coupled receptor signaling pathway; sensory perception of smell; response to stimulus; detection of chemical stimulus involved in sensory perception of smell |
| **Il1rl1** | -1,7 | 0,004422 | negative regulation of T-helper 1 type immune response; signal transduction; negative regulation of cell proliferation; negative regulation of interferon-gamma production; positive regulation of interleukin-5 production; interleukin-33-mediated signaling pathway; positive regulation of macrophage activation; negative regulation of I-kappaB kinase/NF-kappaB signaling; positive regulation of inflammatory response; positive regulation of chemokine secretion; cytokine-mediated signaling pathway |
| **Olfr1325** | -1,69 | 0,015674 | signal transduction; G-protein coupled receptor signaling pathway; sensory perception of smell; response to stimulus; detection of chemical stimulus involved in sensory perception of smell |
| **Pglyrp1** | -1,69 | 0,047673 | pattern recognition receptor signaling pathway; immune system process; apoptotic process; peptidoglycan catabolic process; detection of bacterium; negative regulation of interferon-gamma production; negative regulation of natural killer cell differentiation involved in immune response; defense response to bacterium; growth of symbiont in host; innate immune response; negative regulation of inflammatory response; defense response to Gram-positive bacterium |
| **H2-Ab1** | -1,67 | 0,010355 | B cell affinity maturation; immune system process; immunoglobulin production involved in immunoglobulin mediated immune response; humoral immune response mediated by circulating immunoglobulin; antigen processing and presentation of peptide or polysaccharide antigen via MHC class II; positive regulation of antigen processing and presentation; positive regulation of T-helper 1 type immune response.. |
| **Cma1** | -1,67 | 0,003534 | proteolysis; positive regulation of angiogenesis; interleukin-1 beta biosynthetic process; peptide metabolic process; midbrain development; cellular response to glucose stimulus |
| **H2-Aa** | -1,66 | 0,002109 | immune system process; antigen processing and presentation of peptide or polysaccharide antigen via MHC class II; immune response; antigen processing and presentation; antigen processing and presentation of exogenous peptide antigen via MHC class II; response to interferon-gamma; negative regulation of T cell proliferation; positive regulation of T cell differentiation; antigen processing and presentation of peptide antigen; antigen processing and presentation of peptide antigen via MHC class I |
| **Cd22** | -1,66 | 0,008805 | cell adhesion; cell surface receptor signaling pathway |
| **H2-Eb2; H2-Eb1** | -1,65 | 0,022092 | immune system process; antigen processing and presentation of peptide or polysaccharide antigen via MHC class II; immune response; antigen processing and presentation; antigen processing and presentation of exogenous peptide antigen via MHC class II; response to interferon-gamma |
| **Il2** | -1,6 | 0,02122 |  |
| **Pik3ap1** | -1,6 | 0,004017 | positive regulation of phosphatidylinositol 3-kinase signaling; negative regulation of toll-like receptor signaling pathway; positive regulation of toll-like receptor signaling pathway; toll-like receptor 2 signaling pathway; toll-like receptor 4 signaling pathway; toll-like receptor 7 signaling pathway; toll-like receptor 9 signaling pathway; regulation of I-kappaB kinase/NF-kappaB signaling; regulation of MAPK cascade; regulation of inflammatory response |
| **Ccl3** | -1,56 | 0,002094 |  |
| **S1pr3** | -1,55 | 0,000883 | cytokine production; inflammatory response; signal transduction; G-protein coupled receptor signaling pathway; adenylate cyclase-inhibiting G-protein coupled receptor signaling pathway; regulation of interleukin-1 beta production; sphingosine-1-phosphate signaling pathway |
| **Olfr399** | -1,55 | 0,032205 | signal transduction; G-protein coupled receptor signaling pathway; sensory perception of smell; response to stimulus; detection of chemical stimulus involved in sensory perception of smell |
| **Olfr165** | -1,54 | 0,041756 | signal transduction; G-protein coupled receptor signaling pathway; sensory perception of smell; response to stimulus; detection of chemical stimulus involved in sensory perception of smell |
| **Il13** | -1,53 | 0,000331 | microglial cell activation; positive regulation of immunoglobulin production; immune response; regulation of proton transport; positive regulation of B cell proliferation; positive regulation of connective tissue growth factor production; negative regulation of NAD(P)H oxidase activity; response to nicotine; positive regulation of tyrosine phosphorylation of Stat6 protein; positive regulation of macrophage activation.. |
| **Cd24a** | -1,53 | 0,006012 |  |
