# Supplementary material for: Toll-Like Receptor 2 Mediates In Vivo Pro- and Anti-inflammatory Effects of Mycobacterium Tuberculosis and Modulates Autoimmune Encephalomyelitis
Source: Front Immunol. 2016 May 24;7:191. doi: 10.3389/fimmu.2016.00191 (PMC4878199; doi:10.3389/fimmu.2016.00191)
Supplement: Supplementary file 2 [file Table_2.DOCX]

Supplementary Table 2

List of primers used for Quantitative RT-PCR

| **Supplementary Table 2** | |
| --- | --- |
| **Name** | Sequence |
| IFN−γ | 5’-CAGCAACAGCAAGGCGAAAAAGG-3’ |
| IL-17A | 5′-CAGACTACCTCAACCGTTCCAC-3′ |
| IL-10 | 5′-GCTCCTAGAGCTGCGGACT-3′ |
| IL-13 | 5′- AACGGCAGCATGGTATGGAGTG-3′ |
| IL-5 | 5’-CAGCAACAGCAAGGCGAAAAAGG-3’ |
| IL-6 | 5′-ACACATGTTCTCTGGGAAATCGT-3′ |
| TGF-b | 5′-ACCCCCACTGATACGCCTGA-3′ |
| IL-12b | 5′-GAAGCACGGCAGCAGAAT-3′ |
| IL-23 | 5′-CATGGGCTATCAGGGAGTA-3′ |
| FoxP3 | 5′-CCTGGTTGTGAGAAGGTCTTCG-3′ |
| T-bet | 5′-AGCAAGGACGGCGAATGTT-3’ |
| ROR-γT | 5′-CTACTGAGGAGGACAGGGAG-3′ |
| GATA-3 | 5′-CTCGGCCATTCGTACATGGAA-3′ |
| 18S | 5′-CTGCCCTATCAACTTTCGATGG -3′ |
