# Supplementary material for: Toll-Like Receptor 2 Mediates In Vivo Pro- and Anti-inflammatory Effects of Mycobacterium Tuberculosis and Modulates Autoimmune Encephalomyelitis
Source: Front Immunol. 2016 May 24;7:191. doi: 10.3389/fimmu.2016.00191 (PMC4878199; doi:10.3389/fimmu.2016.00191)
Supplement: Supplementary file 4 [file Image_2.PDF]

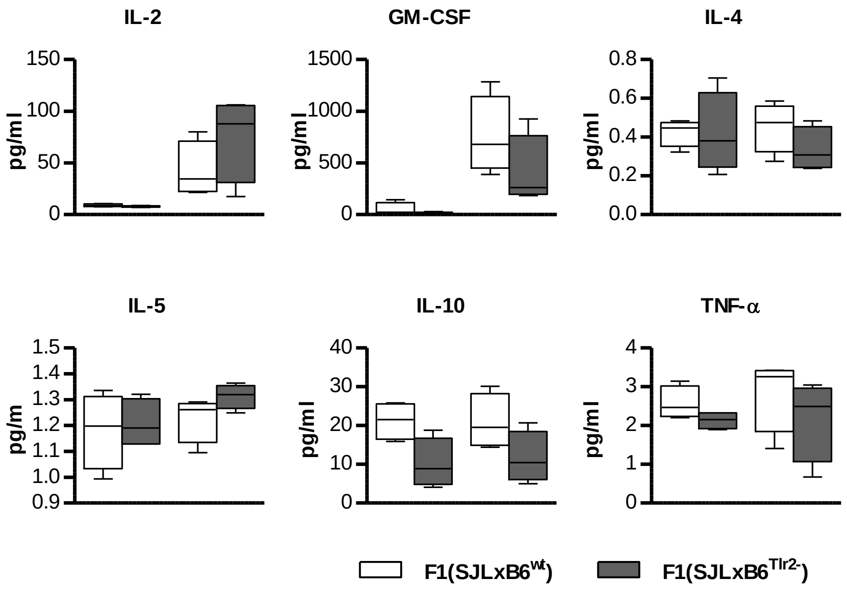

Supplementary Figure 2: Cytokines production quantification. Groups of 6 F1(SJLxB6^{wt}) (open bars) and 6 F1(SJLxB6^{Tlr2-}) (closed bars) mice from two distinct experiments were immunized s.c. with p139 in enriched CFA. Lymph nodes cells were cultured for 18 hours with or without p139 as described. The graphics report the effect of antigen stimulation on the production of cytokines as boxplots. Cytokines quantification was performed by Macsplex.
