# Supplementary material for: Toll-Like Receptor 2 Mediates In Vivo Pro- and Anti-inflammatory Effects of Mycobacterium Tuberculosis and Modulates Autoimmune Encephalomyelitis
Source: Front Immunol. 2016 May 24;7:191. doi: 10.3389/fimmu.2016.00191 (PMC4878199; doi:10.3389/fimmu.2016.00191)
Supplement: Supplementary file 5 [file Image_3.PDF]

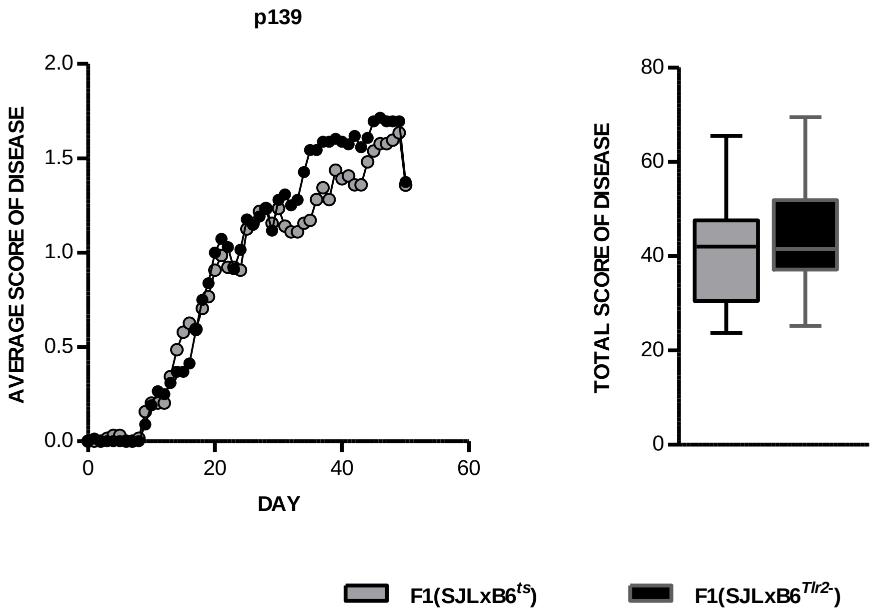

Supplementary Figure 3: Gene dosage of *Tlr2* doesn't modulates severity and course of EAE. Score of disease in 16 F1(SJLxB6^{ts}) mice and 17 F1(SJLxB6^{Tlr2-}) mice. EAE score was evaluated as described in the Materials and Methods. Data reported as the average score are the result of independent experiments; mice were evaluated daily for 50 days in a blind fashion with respect to genotype. Closed symbols and bars report data from F1(SJLxB6^{Tlr2-}) mice (with one copy of Tlr2^{82Ile}); gray symbols and bars report data from F1(SJLxB6^{ts}) mice (with two copies of Tlr2^{82Ile}).
