# Supplementary material for: Toll-Like Receptor 2 Mediates In Vivo Pro- and Anti-inflammatory Effects of Mycobacterium Tuberculosis and Modulates Autoimmune Encephalomyelitis
Source: Front Immunol. 2016 May 24;7:191. doi: 10.3389/fimmu.2016.00191 (PMC4878199; doi:10.3389/fimmu.2016.00191)
Supplement: Supplementary file 3 [file Image_1.PDF]

A

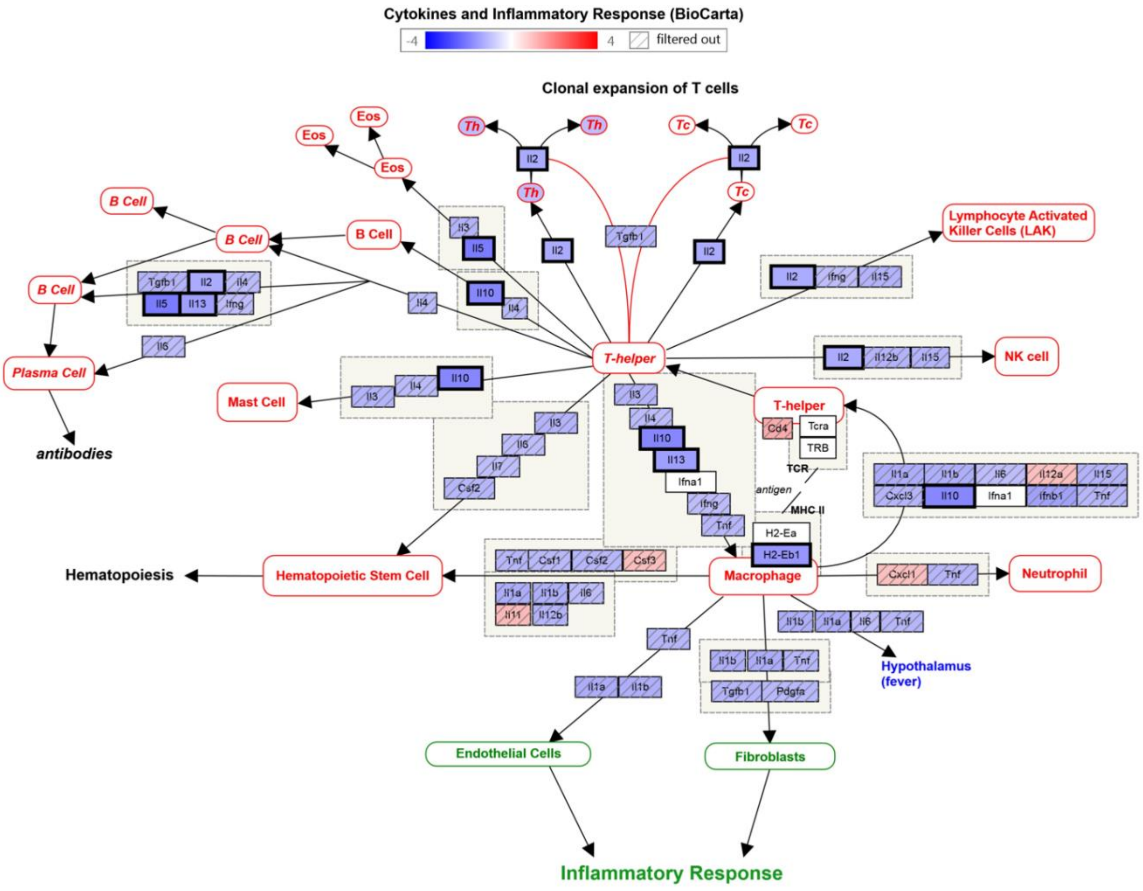

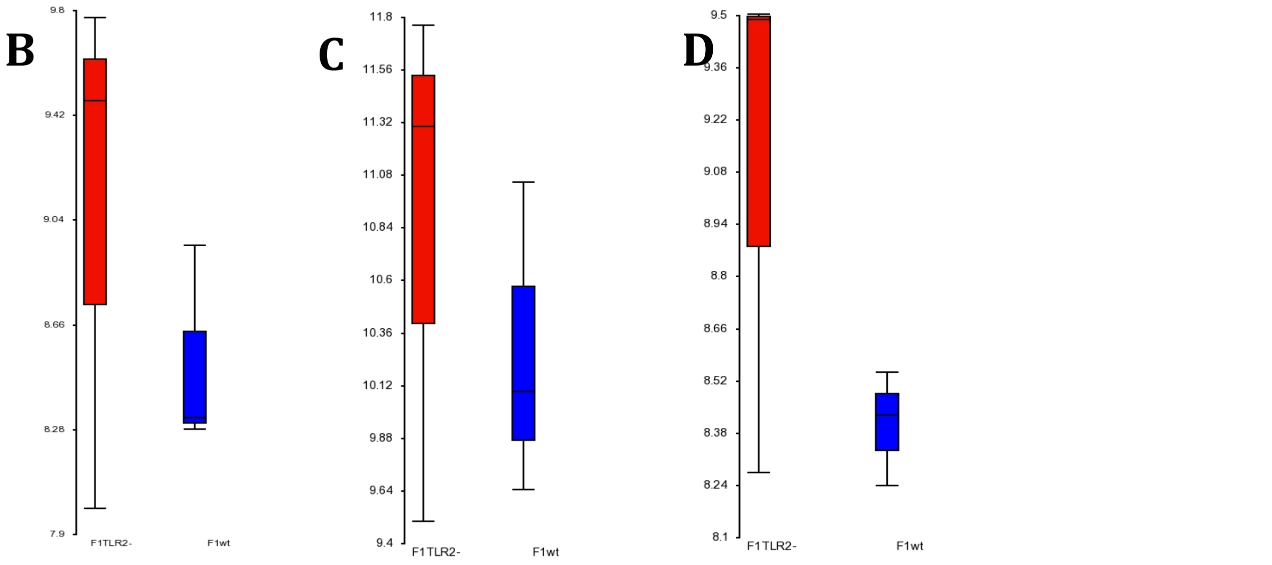

Supplementary Figure 1. **A**: **Cytokines and Inflammatory Response pathway**. The figure shows differentially expressed (in bold format) and filtered out genes. **B-D**: Box plots showing the gene expression pattern (in normalized expression values, see Matherials and Methods) of (B) IL-17f gene, (C) IL-22 gene and (D) Rorc gene in isolated T cells from LN at day 4 after CFA immunization of (F1 (SJLx $B6^{Tlr2-}$) (red) and (F1 ($SJLxB6^{wt}$ Blue) mice. Data from three biological replicates are shown.
